# Using sensitivity equations for computing gradients of the FOCE and FOCEI approximations to the population likelihood

**DOI:** 10.1007/s10928-015-9409-1

**Published:** 2015-03-24

**Authors:** Joachim Almquist, Jacob Leander, Mats Jirstrand

**Affiliations:** 1 Fraunhofer-Chalmers Centre, Chalmers Science Park, 41288 Gothenburg, Sweden; 2 Systems and Synthetic Biology, Department of Biology and Biological Engineering, Chalmers University of Technology, 41296 Gothenburg, Sweden; 3 Department of Mathematical Sciences, Chalmers University of Technology and University of Gothenburg, 41296 Gothenburg, Sweden

**Keywords:** Nonlinear mixed effects modeling, First order conditional estimation (FOCE), Sensitivity equations

## Abstract

The first order conditional estimation (FOCE) method is still one of the parameter estimation workhorses for nonlinear mixed effects (NLME) modeling used in population pharmacokinetics and pharmacodynamics. However, because this method involves two nested levels of optimizations, with respect to the empirical Bayes estimates and the population parameters, FOCE may be numerically unstable and have long run times, issues which are most apparent for models requiring numerical integration of differential equations. We propose an alternative implementation of the FOCE method, and the related FOCEI, for parameter estimation in NLME models. Instead of obtaining the gradients needed for the two levels of quasi-Newton optimizations from the standard finite difference approximation, gradients are computed using so called sensitivity equations. The advantages of this approach were demonstrated using different versions of a pharmacokinetic model defined by nonlinear differential equations. We show that both the accuracy and precision of gradients can be improved extensively, which will increase the chances of a successfully converging parameter estimation. We also show that the proposed approach can lead to markedly reduced computational times. The accumulated effect of the novel gradient computations ranged from a 10-fold decrease in run times for the least complex model when comparing to forward finite differences, to a substantial 100-fold decrease for the most complex model when comparing to central finite differences. Considering the use of finite differences in for instance NONMEM and Phoenix NLME, our results suggests that significant improvements in the execution of FOCE are possible and that the approach of sensitivity equations should be carefully considered for both levels of optimization.

## Introduction

Nonlinear mixed effects (NLME) models are suitable in situations where sparse time-series data is collected from a population of individuals exhibiting inter-individual variability [10]. This property has rendered NLME models popular in both pharmacokinetics and pharmacodynamics, and several public and commercial software packages have been developed for performing NLME modeling within these fields [13]. These modeling softwares include the well-known NONMEM [5], which was the first program to be developed and still is one of the most widely used, but also a number of other programs such as Phoenix NLME [21] and Monolix [15]. A core part of their functionality consist of various methods for addressing the problem of parameter estimation in NLME models, and several studies have been devoted to describing and comparing different aspects of these methods [4, 8, 9, 11, 12, 22].

The “mixed effects” in NLME refers to the fact that these models contain both fixed effect parameters, having the same value for all individuals, and random effect parameters, whose value differ from one individual to another and whose distribution in the population is determined by some statistical model. A common approach to the parameter estimation problem in NLME models is based on maximizing the so called population likelihood. The population likelihood is a function of the fixed effect parameters only, and it is obtained by marginalizing out the random effects from the joint distribution of data and random effects. However, the integral required for the marginalization lacks a closed-form solution for all realistic problems. Because of this, maximum likelihood parameter estimation for NLME models revolves around different numerical approximation methods for computing this integral. One of the main approaches for tackling the problem is a class of related methods based on the so called Laplacian approximation [25]. It includes the popular and widely used first order conditional estimation (FOCE) method, which is a special case of the closely related FOCE with interaction (FOCEI). With the FOCE and FOCEI methods, the approximation of the integral involves a Taylor expansion around the values of the random effect parameters that maximize the joint distribution. This means that one optimization problem per individual has to be solved for every evaluation of the approximated population likelihood. Since the aim is to maximize the (approximated) population likelihood, which constitutes the original optimization problem, conditional estimation methods such as FOCE produce a parameter estimation problem involving two nested layers of optimizations. For some NLME parameter estimation problems this results in long execution times, and in difficulties with numerical precision making the optimizations unstable and limiting the precision of estimates and the ability of obtaining confidence intervals. These issues are particularly pronounced for models that are formulated by systems of differential equations which are lacking analytical solutions [4, 7, 8].

The optimization problems resulting from the FOCE and FOCEI approximations, and other closely related approximations, are typically solved using gradient-based optimization methods such as the Broyden–Fletcher–Goldfarb–Shanno (BFGS) quasi-Newton method [20]. For problems where analytical expressions for the function and its gradient are not available, it is common that gradients are computed by finite difference approximations. We instead propose another approach for determining the gradient of the FOCE and FOCEI approximations of the population likelihood. Our approach is based on formally differentiating the likelihoods used at the two levels of optimization, and computing the required derivatives of the model state variables using so called sensitivity equations. The proposed approach for computing gradients is readily applicable for the inner level of the nested optimization problem. However, we also derive the necessary theory for computing gradients through the approach of sensitivity equations at the outer level optimization. This step is the more challenging, and requires that sensitivities up to second order of the state variables with respect to the parameters and random parameters are obtained. Being able to compute the gradient of the FOCE or FOCEI approximations of the population likelihood using the approach introduced in this paper is a great advantage as it circumvents the need for repeatedly having to solve the inner level optimization problem for obtaining the outer level gradients from a finite difference approximation.

This paper is organized in the following way. First, the mathematical theory is introduced. Here we recapitulate NLME models based on differential equations, including the formulation of the population likelihood and its approximations, as well as derive expressions for both the gradients of the individual joint log-likelihoods with respect to the random effect parameters, used for the inner level optimization problems, and the gradient of the approximate population likelihood with respect to the fixed effect parameters, used for the outer level optimization problem. Then, we apply the sensitivity approach for computing the gradients for different versions of a benchmark model. Compared to the finite difference approximation, the proposed approach leads to both higher precision and better accuracy of the gradient, as well as decreased computational times. Finally, the presented results are discussed and possible future extensions are outlined.

## Theory

Various definitions and results from matrix calculus are used in the derivations of this section. These can be found in the "Appendix 1" section.

### The nonlinear mixed effects model

Consider a population of $N$ subjects and let the $i$th individual be described by the dynamical system

1 $$\begin{aligned} \begin{aligned} \frac{d{\mathbf{x}}_i(t)}{dt}&= {\mathbf{f}}\big ({\mathbf{x}}_i(t),t,{\mathbf{Z}}_i(t),\varvec{\theta },\varvec{\eta }_i\big )\\ {\mathbf{x}}_i(t_0)&= {\mathbf{x}}_{0i}\big ({\mathbf{Z}}_i(t_0),\varvec{\theta },\varvec{\eta }_i\big ), \end{aligned} \end{aligned}$$

where ${\mathbf{x}}_i(t)$ is a set of state variables, which for instance could be used to describe a drug concentration in one or more compartments, and where ${\mathbf{Z}}_i(t)$ is a set of possibly time dependent covariates, $\varvec{\theta }$ a set of fixed effects parameters, and $\varvec{\eta }_i$ a set of random effect parameters which are multivariate normally distributed with zero mean and covariance $\varvec{{\Omega }}$. The covariance matrix $\varvec{{\Omega }}$ is in general unknown and will therefore typically contain parameters subject to estimation. These parameters will for convenience of notation be included in the fixed effect parameter vector $\varvec{\theta }$. Fixed effects parameters will hence be used to refer to all parameters that are not random, not being limited for parameters appearing in the model differential equations. A model for the $j$th observation of the $i$th individual at time $t_{j_i}$ is defined by

2 $$\begin{aligned} {\mathbf{y}}_{ij} = {\mathbf{h}}\big ({\mathbf{x}}_{ij},t_{j_i},{\mathbf{Z}}_i(t_{j_i}),\varvec{\theta },\varvec{\eta }_i\big ) + {\mathbf{e}}_{ij}, \end{aligned}$$

where

3 $$\begin{aligned} {\mathbf{e}}_{ij} \in N\Big (\varvec{0},{\mathbf{R}}_{ij}\big ({\mathbf{x}}_{ij},t_{j_i},{\mathbf{Z}}_i(t_{j_i}),\varvec{\theta },\varvec{\eta }_i\big )\Big ), \end{aligned}$$

and where the index notation $ij$ is used as a short form for denoting the $i$th individual at the $j$th observation. Note that any fixed effect parameters of the observational model are included in $\varvec{\theta }$. Furthermore, we let the expected value of the discrete-time observation model be denoted by

4 $$\begin{aligned} \hat {\mathbf{y}}_{ij} = {\mathbf{E}}\big [{\mathbf{y}}_{ij}\big ]. \end{aligned}$$

### The population likelihood

Given a set of experimental observations, ${\mathbf{d}}_{ij}$, for the individuals $i=1, \ldots , N$ at the time points $t_{j_i}$, where $j=1, \dots , n_{i}$, we define the residuals

5 $$\begin{aligned} \varvec{\epsilon }_{ij} = {\mathbf{d}}_{ij} - \hat{\mathbf{y}}_{ij}, \end{aligned}$$

and write the population likelihood

6 $$\begin{aligned} L(\varvec{\theta }) = \prod _{i=1}^N \int p_1\big ({\mathbf{d}}_{i}|\varvec{\theta },\varvec{\eta }_i\big )p_2\big (\varvec{{\eta }}_i|\varvec{\theta }\big )\,d\varvec{\eta }_i, \end{aligned}$$

where

7 $$\begin{aligned} p_1\big ({\mathbf{d}}_i|\varvec{\theta },\varvec{\eta }_i\big ) = \prod _{j=1}^{n_i} \frac{\exp \big (-\frac{1}{2}\varvec{\epsilon }_{ij}^T{\mathbf{R}}_{ij}^{-1}\varvec{\epsilon }_{ij}\big )}{\sqrt{\det \big (2\pi {\mathbf{R}}_{ij}\big )}} \end{aligned}$$

and

8 $$\begin{aligned} p_2\big (\varvec{\eta }_i|\varvec{\theta }\big ) = \frac{\exp \big (-\frac{1}{2}\varvec{\eta }_i^T\varvec{{\Omega }}^{-1}\varvec{\eta }_i\big )}{\sqrt{\det \big (2\pi \varvec{{\Omega }}\big )}}, \end{aligned}$$

and where ${\mathbf{d}}_{i}$ is used to denote the collection of data from all time points for the $i$th individual.

### The FOCE and FOCEI approximations

The marginalization with respect to $\varvec{\eta }_i$ in Eq. 6 does not have a closed form solution. By writing Eq. 6 on the form

9 $$\begin{aligned} L(\varvec{\theta }) = \prod _{i=1}^N \int \exp (l_i)\,d\varvec{\eta }_i, \end{aligned}$$

where the individual joint log-likelihoods are

10 $$\begin{aligned} \begin{aligned} l_i&= -\frac{1}{2}\sum _{j=1}^{n_i} \left( \varvec{\epsilon }_{ij}^T {\mathbf{R}}_{ij}^{-1}\varvec{\epsilon }_{ij} + \log \det \big (2\pi {\mathbf{R}}_{ij}\big ) \right) \\&\quad -\frac{1}{2} \varvec{\eta }_i^T \varvec{{\Omega }}^{-1}\varvec{\eta }_i-\frac{1}{2}\log \det \big (2\pi \varvec{{\Omega }}\big ), \end{aligned} \end{aligned}$$

a closed form solution can be obtained by approximating the function $l_i$ with a second order Taylor expansion with respect to $\varvec{\eta }_i$. This is the well-known Laplacian approximation. Furthermore, we let the point around which the Taylor expansion is done to be conditioned on the $\varvec{\eta }_i$ maximizing $l_i$, here denoted by $\varvec{\eta }_i^*$; I.e., the expansion is done at the mode of the posterior distribution. Thus, the approximate population likelihood, $L_{L}$, becomes

11 $$\begin{aligned} L(\varvec{\theta }) \approx L_{L}(\varvec{\theta }) = \prod _{i=1}^N \left( \exp \big (l_i(\varvec{\eta }_i^*)\big ) \det \left[ \frac{-\mathrm {\Delta } l_i(\varvec{\eta }_i^*)}{2\pi } \right] ^{-\frac{1}{2}} \right) . \end{aligned}$$

Here, the Hessian $\mathrm {\Delta } l_i(\varvec{\eta }_i^*)$ is obtained by first differentiating $l_i$ twice with respect to $\varvec{\eta }_i$, and evaluating at $\varvec{\eta }_i^*$. If we let $\eta _{ik}$ denote the $k$th component of $\varvec{\eta }_i$, we have

12 $$\begin{aligned} \begin{aligned} \frac{d l_{i}}{d \eta _{ik}} =&- \frac{1}{2} \sum _{j=1}^{n_{i}} \Bigg ( 2 \varvec{\epsilon }_{ij}^T {\mathbf{R}}_{ij}^{-1} \frac{d \varvec{\epsilon }_{ij}}{d \eta _{ik}} - \varvec{\epsilon }_{ij}^T {\mathbf{R}}_{ij}^{-1} \frac{d {\mathbf{R}}_{ij}}{d \eta _{ik}} {\mathbf{R}}_{ij}^{-1} \varvec{\epsilon }_{ij}\\&\quad + {{\mathrm{tr}}}\left[ \mathrm {{\mathbf{R}}_{ij}^{-1}} \frac{d {\mathbf{R}}_{ij}}{d \eta _{ik}} \right] \Bigg ) - \varvec{\eta }_i^T \varvec{{\Omega }}^{-1} \frac{d \varvec{\eta }_i}{d \eta _{ik}}. \end{aligned} \end{aligned}$$

Differentiating component-wise again, now with respect to the $l$th component of $\varvec{\eta }_i$, we get the elements of the Hessian

13 $$\begin{aligned} \begin{aligned} \frac{d^2 l_{i}}{d \eta _{ik} d \eta _{il}} =&- \frac{1}{2} \sum _{j=1}^{n_{i}} \Bigg ( 2 \frac{d \varvec{\epsilon }_{ij}^T}{d \eta _{il}} {\mathbf{R}}_{ij}^{-1} \frac{d \varvec{\epsilon }_{ij}}{d \eta _{ik}} - 2 \varvec{\epsilon }_{ij}^T {\mathbf{R}}_{ij}^{-1} \frac{d {\mathbf{R}}_{ij}}{d \eta _{il}} {\mathbf{R}}_{ij}^{-1} \frac{d \varvec{\epsilon }_{ij}}{d \eta _{ik}}\\&\quad + 2 \varvec{\epsilon }_{ij}^T {\mathbf{R}}_{ij}^{-1} \frac{d^2 \varvec{\epsilon }_{ij}}{d \eta _{ik} d \eta _{il}} - \varvec{\epsilon }_{ij}^T {\mathbf{R}}_{ij}^{-1} \frac{d^2 {\mathbf{R}}_{ij}}{d \eta _{ik} d \eta _{il}} {\mathbf{R}}_{ij}^{-1} \varvec{\epsilon }_{ij}\\&\quad + 2 \varvec{\epsilon }_{ij}^T {\mathbf{R}}_{ij}^{-1} \frac{d {\mathbf{R}}_{ij}}{d \eta _{ik}} {\mathbf{R}}_{ij}^{-1} \frac{d {\mathbf{R}}_{ij}}{d \eta _{il}} {\mathbf{R}}_{ij}^{-1} \varvec{\epsilon }_{ij} - 2 \varvec{\epsilon }_{ij}^T {\mathbf{R}}_{ij}^{-1} \frac{d {\mathbf{R}}_{ij}}{d \eta _{ik}} {\mathbf{R}}_{ij}^{-1} \frac{d \varvec{\epsilon }_{ij}}{d \eta _{il}}\\&- {{\mathrm{tr}}}\left[ {\mathbf{R}}_{ij}^{-1} \frac{d {\mathbf{R}}_{ij}}{d \eta _{il}} {\mathbf{R}}_{ij}^{-1} \frac{d {\mathbf{R}}_{ij}}{d \eta _{ik}}\right] + {{\mathrm{tr}}}\left[ {\mathbf{R}}_{ij}^{-1} \frac{d^2 {\mathbf{R}}_{ij}}{d \eta _{ik} d \eta _{il}} \right] \Bigg )\\&- \frac{d \varvec{\eta }_i^T}{d \eta _{il}} \varvec{{\Omega }}^{-1} \frac{d \varvec{\eta }_i}{d \eta _{ik}}, \end{aligned} \end{aligned}$$

where the last term is really just the $kl$th element of $\varvec{{\Omega }}^{-1}$, $\mathrm {\Omega }^{-1}_{kl}$. The expression for the elements of the Hessian may be approximated in different ways, with the main purpose of avoiding the need for computing the costly second order derivatives. We apply a first order approximation, where terms containing second order derivatives are ignored, and write the elements of the approximate Hessian, ${\mathbf{H}}_i$, as

14 $$\begin{aligned} \mathrm {H}_{ikl} = - \frac{1}{2} \sum _{j=1}^{n_{i}} \Bigg ( {\mathbf{a}}_l \, {\mathbf{B}} \, {\mathbf{a}}^T_k + {{\mathrm{tr}}}\left[ - {\mathbf{c}}_l \, {\mathbf{c}}_k \right] \Bigg ) - \mathrm {\Omega }_{kl}^{-1}, \end{aligned}$$

where

15 $$\begin{aligned} {\mathbf{a}}_k = \left( \frac{d \varvec{\epsilon }_{ij}^T}{d \eta _{ik}} - \varvec{\epsilon }_{ij}^T {\mathbf{R}}_{ij}^{-1} \frac{d {\mathbf{R}}_{ij}}{d \eta _{ik}} \right) ,\end{aligned}$$

16 $$\begin{aligned} {\mathbf{B}} = 2{\mathbf{R}}_{ij}^{-1}, \end{aligned}$$

and

17 $$\begin{aligned} {\mathbf{c}}_k = {\mathbf{R}}_{ij}^{-1} \frac{d {\mathbf{R}}_{ij}}{d \eta _{ik}}. \end{aligned}$$

This variant of the Laplacian approximation of the population likelihood is known as the first order conditional estimation with interaction (FOCEI) method. The closely related first order conditional estimation (FOCE) method is obtained by ignoring the dependence of the residual covariance matrix on the random effect parameters. The rationale for excluding the second order terms is that their expected values are zero for an appropriate model, as shown in the "Appendix 2" section. The Appendix also shows how the Hessian may be slightly further simplified, using similar arguments, to arrive at the variant of FOCE used in NONMEM. Those additional simplifications are however of relatively little importance from a computational point of view, since the components needed to evaluate these Hessian terms have to be provided for the remaining part of the Hessian anyway. We will therefore restrict the Hessian simplification by expectation to the second order terms only. Furthermore, we will from now on for convenience consider the logarithm of the FOCEI approximation to the population likelihood, $L_{F}$,

18 $$\begin{aligned} \log L(\varvec{\theta }) \approx \log L_{F}(\varvec{\theta }) = \sum _{i=1}^N \left( l_i(\varvec{\eta }_i^*) -\frac{1}{2} \log \det \left[ \frac{-{\mathbf{H}}_i(\varvec{\eta }_i^*)}{2\pi } \right] \right) . \end{aligned}$$

### Gradient of the individual joint log-likelihood with respect to the random effect parameters

We now turn to the computation of the gradient of the individual joint log-likelihoods, $l_i(\varvec{\eta }_i)$, with respect to the random effect parameters, $\varvec{\eta }_i$, using the approach of sensitivity equations. Consider the differentiation done in Eq. 12. Given values of $\varvec{\theta }$ and $\varvec{\eta }_i$, the quantities $\varvec{\epsilon }_{ij}$, ${\mathbf{R}}_{ij}$, and $\varvec{{\Omega }}$ can be obtained by solving the model equations. However, we additionally need to determine $d \varvec{\epsilon }_{ij} / d \eta _{ik}$ and $d {\mathbf{R}}_{ij} / d \eta _{ik}$. Expanding the total derivative of these quantities we see that

19 $$\begin{aligned} \frac{d \varvec{\epsilon }_{ij}}{d \eta _{ik}} = \frac{d \big ({\mathbf{d}}_{ij} - \hat{\mathbf{y}}_{ij}\big )}{d \eta _{ik}} = - \left( \frac{\partial {\mathbf{h}}}{\partial \eta _{ik}} + \frac{\partial {\mathbf{h}}}{\partial {\mathbf{x}}_{ij}} \frac{d {\mathbf{x}}_{ij}}{d \eta _{ik}} \right) , \end{aligned}$$

and

20 $$\begin{aligned} \frac{d {\mathbf{R}}_{ij}}{d \eta _{ik}} = \frac{\partial {\mathbf{R}}_{ij}}{\partial \eta _{ik}} + \frac{\partial {\mathbf{R}}_{ij}}{\partial {\mathbf{x}}_{ij}} \frac{d {\mathbf{x}}_{ij}}{d \eta _{ik}}. \end{aligned}$$

The derivatives of ${\mathbf{h}}$ and ${\mathbf{R}}_{ij}$ are readily obtained since these expressions are given explicitly by the model formulation. In contrast, the derivative of the state variables, ${\mathbf{x}}_{ij}$, are not directly available but can be computed from the so called sensitivity equations. The sensitivity equations are a set of differential equations which are derived by differentiating the original system of differential equations (and the corresponding initial conditions) with respect to each random effect parameter $\eta _{ik}$,

21 $$\begin{aligned} \begin{aligned} \frac{d}{d t} \left( \frac{d {\mathbf{x}}_{i}}{d \eta _{ik}} \right)&= \frac{\partial {\mathbf{f}}}{\partial \eta _{ik}} + \frac{\partial {\mathbf{f}}}{\partial {\mathbf{x}}_{i}} \left( \frac{d {\mathbf{x}}_{i}}{d \eta _{ik}} \right) \\ \left( \frac{d {\mathbf{x}}_i}{d \eta _{ik}} \right) (t_0)&= \frac{\partial {\mathbf{x}}_{0i}}{\partial \eta _{ik}}. \end{aligned} \end{aligned}$$

The solution to the sensitivity equations can be used to evaluate the derivatives in Eqs. 19 and 20, which in turn are needed for the gradient of the individual joint log-likelihoods. Importantly, these derivatives are also used for computing the approximate Hessian, Eq. 14, appearing in the approximate population log-likelihood.

In the unusual event that one or more of the random effect parameters only appear in the observational model, all sensitivities of the state variables with respect to those parameters are trivially zero. Note also that the sensitivity equations for all but trivial models involve the original state variables, which means that the original system of differential equations has to be solved simultaneously. Thus, if there are $q$ non-trivial sensitivities and $n$ state variables, the total number of differential equations that has to be solved in order to be able to compute $l_i$ and $d l_i / d \varvec{\eta }_i$ for each individual is

22 $$\begin{aligned} n (1 + q). \end{aligned}$$

### Gradient of the approximate population log-likelihood with respect to the fixed effect parameters

We now derive the expression for the gradient of the approximate population log-likelihood, $\log L_{F}(\varvec{\theta })$, with respect to the parameter vector $\varvec{\theta }$. Differentiating $\log L_{F}$ with respect to the $m$th element of $\varvec{\theta }$ gives

23 $$\begin{aligned} \frac{\log L_{F}}{d \theta _m} = \sum _{i=1}^N \left( \frac{d l_i(\varvec{\eta }_i^*)}{d \theta _m} -\frac{1}{2} {{\mathrm{tr}}}\left[ {\mathbf{H}}_i^{-1}(\varvec{\eta }_i^*) \frac{d {\mathbf{H}}_i(\varvec{\eta }_i^*)}{d \theta _m} \right] \right) . \end{aligned}$$

Here it must be emphasized that all derivatives with respect to components of the parameter vector $\varvec{\theta }$ are taken *after* replacing $\varvec{\eta }_i$ with $\varvec{\eta }_i^*$. This is critical since $\varvec{\eta }_i^*$ is an implicit function of theta, $\varvec{\eta }_i^*=\varvec{\eta }_i^*(\varvec{\theta })$. In other words, we have to account for the fact that the $\varvec{\eta }_i$ maximizing the individual joint log-likelihood changes as $\varvec{\theta }$ changes.

To determine the total derivatives with respect to components of the parameter vector $\varvec{\theta }$ we will be needing the following result. Consider a function ${\mathbf{v}}$ which may depend directly on the parameters $\varvec{\theta }$ and $\varvec{\eta }_i$, and on the auxiliary function ${\mathbf{w}}$ representing any indirect dependencies of these parameters,

24 $$\begin{aligned} {\mathbf{v}} = {\mathbf{v}} \big ({\mathbf{w}}(\varvec{\theta },\varvec{\eta }_i),\varvec{\theta },\varvec{\eta }_i\big ). \end{aligned}$$

We furthermore introduce the function ${\mathbf{z}}$ to denote the evaluation of ${\mathbf{v}}$ at $\varvec{\eta }_i=\varvec{\eta }_i^*(\varvec{\theta })$,

25 $$\begin{aligned} {\mathbf{z}} = {\mathbf{z}} \big ({\mathbf{w}}(\varvec{\theta },\varvec{\eta }_i^*(\varvec{\theta })),\varvec{\theta },\varvec{\eta }_i^*(\varvec{\theta })\big ) = \left. {\mathbf{v}} \right| _{\varvec{\eta }_i=\varvec{\eta }_i^*(\varvec{\theta })}. \end{aligned}$$

Separating the complete dependence of ${\mathbf{z}}$ on $\varvec{\theta }$ into partial dependencies we get that

26 $$\begin{aligned} \begin{aligned}&\frac{d }{d \varvec{\theta }} \left( \left. {\mathbf{v}} \right| _{\varvec{\eta }_i=\varvec{\eta }_i^*(\varvec{\theta })} \right) = \frac{d {\mathbf{z}}}{d \varvec{\theta }}\\&\quad = \frac{\partial {\mathbf{z}}}{\partial {\mathbf{w}}}\frac{d {\mathbf{w}}}{d \varvec{\theta }} + \frac{\partial {\mathbf{z}}}{\partial \varvec{\theta }} + \frac{\partial {\mathbf{z}}}{\partial \varvec{\eta }_i^*}\frac{d \varvec{\eta }_i^*}{d \varvec{\theta }}\\&\quad = \frac{\partial {\mathbf{z}}}{\partial {\mathbf{w}}}\frac{\partial {\mathbf{w}}}{\partial \varvec{\theta }} + \frac{\partial {\mathbf{z}}}{\partial {\mathbf{w}}}\frac{\partial {\mathbf{w}}}{\partial \varvec{\eta }_i^*}\frac{d \varvec{\eta }_i^*}{d \varvec{\theta }} + \frac{\partial {\mathbf{z}}}{\partial \varvec{\theta }} + \frac{\partial {\mathbf{z}}}{\partial \varvec{\eta }_i^*}\frac{d \varvec{\eta }_i^*}{d \varvec{\theta }}\\&\quad = \frac{\partial {\mathbf{z}}}{\partial {\mathbf{w}}}\frac{\partial {\mathbf{w}}}{\partial \varvec{\theta }} + \frac{\partial {\mathbf{z}}}{\partial \varvec{\theta }} + \frac{d {\mathbf{z}}}{d \varvec{\eta }_i^*}\frac{d \varvec{\eta }_i^*}{d \varvec{\theta }}\\&\quad = \frac{\partial }{\partial {\mathbf{w}}} \left( \left. {\mathbf{v}} \right| _{\varvec{\eta }_i=\varvec{\eta }_i^*(\varvec{\theta })} \right) \frac{\partial {\mathbf{w}}}{\partial \varvec{\theta }} +\frac{\partial }{\partial \varvec{\theta }} \left( \left. {\mathbf{v}} \right| _{\varvec{\eta }_i=\varvec{\eta }_i^*(\varvec{\theta })} \right) + \frac{d}{d \varvec{\eta }_i^*} \left( \left. {\mathbf{v}} \right| _{\varvec{\eta }_i=\varvec{\eta }_i^*(\varvec{\theta })} \right) \frac{d \varvec{\eta }_i^*}{d \varvec{\theta }}\\&\quad = \left. \left( \frac{\partial {\mathbf{v}}}{\partial {\mathbf{w}}} \frac{\partial {\mathbf{w}}}{\partial \varvec{\theta }} \right) \right| _{\varvec{\eta }_i=\varvec{\eta }_i^*(\varvec{\theta })} +\left. \left( \frac{\partial {\mathbf{v}}}{\partial \varvec{\theta }} \right) \right| _{\varvec{\eta }_i=\varvec{\eta }_i^*(\varvec{\theta })} + \left. \left( \frac{d {\mathbf{v}}}{d \varvec{\eta }_i} \right) \right| _{\varvec{\eta }_i=\varvec{\eta }_i^*(\varvec{\theta })} \frac{d \varvec{\eta }_i^*}{d \varvec{\theta }}\\&\quad = \left. \frac{d {\mathbf{v}}}{d \varvec{\theta }} \right| _{\varvec{\eta }_i=\varvec{\eta }_i^*(\varvec{\theta })} + \left. \frac{d {\mathbf{v}}}{d \varvec{\eta }_i} \right| _{\varvec{\eta }_i=\varvec{\eta }_i^*(\varvec{\theta })} \frac{d \varvec{\eta }_i^*}{d \varvec{\theta }}. \end{aligned} \end{aligned}$$

Thus, the total derivative with respect to $\varvec{\theta }$ after insertion of $\varvec{\eta }_i^*$ is equal to the sum of total derivatives with respect to $\varvec{\theta }$ and $\varvec{\eta }_i$ before insertion of $\varvec{\eta }_i^*$, where the second derivative is multiplied with the sensitivity of the random effect optimum with respect to the parameters $\varvec{\theta }$. It is straightforward to see that this result holds also when differentiating functions that only exhibit a subset of the possible direct and indirect dependencies of Eq. 24, for instance functions with just an indirect dependence on the two kind of parameters.

Applying the results from Eq. 26 to the first term within the summation of Eq. 23, we have that

27 $$\begin{aligned} \frac{d l_i(\varvec{\eta }_i^*)}{d \theta _m} = \left. \frac{d l_i(\varvec{\eta }_i)}{d \theta _m} \right| _{\varvec{\eta }_i=\varvec{\eta }_i^*(\varvec{\theta })} \,+\, \left. \frac{d l_i(\varvec{\eta }_i)}{d \varvec{\eta }_i} \right| _{\varvec{\eta }_i=\varvec{\eta }_i^*(\varvec{\theta })} \frac{d \varvec{\eta }_i^*}{d \theta _m}. \end{aligned}$$

However, since $d l_i / d \varvec{\eta }_i$ evaluated at $\varvec{\eta }_i^*$ is zero by definition, the second term of the right hand side of Eq. 27 disappears and

28 $$\begin{aligned} \begin{aligned} \frac{d l_i(\varvec{\eta }_i^*)}{d \theta _m} = \left. \frac{d l_i(\varvec{\eta }_i)}{d \theta _m} \right| _{\varvec{\eta }_i=\varvec{\eta }_i^*(\varvec{\theta })}&= \Bigg [ - \frac{1}{2} \sum _{j=1}^{n_{i}} \Bigg ( 2 \varvec{\epsilon }_{ij}^{T} {\mathbf{R}}_{ij}^{-1} \frac{d \varvec{\epsilon }_{ij}}{d \theta _m}\\&\quad - \varvec{\epsilon }_{ij}^{T} {\mathbf{R}}_{ij}^{-1} \frac{d {\mathbf{R}}_{ij}}{d \theta _m} {\mathbf{R}}_{ij}^{-1} \varvec{\epsilon }_{ij} + {{\mathrm{tr}}}\left[ \mathrm {{\mathbf{R}}_{ij}^{-1}} \frac{d {\mathbf{R}}_{ij}}{d \theta _m} \right] \Bigg )\\&\quad + \frac{1}{2} \varvec{\eta }_i^T \, \varvec{{\Omega }}^{-1} \frac{d \varvec{{\Omega }}}{d \theta _m} {\varvec{\Omega }}^{-1} \varvec{\eta }_i - \frac{1}{2} {{\mathrm{tr}}}\left[ \varvec{{\Omega }}^{-1} \frac{d \varvec{{\Omega }}}{d \theta _m} \right] \Bigg ]_{\varvec{\eta }_i=\varvec{\eta }_i^*(\varvec{\theta })}. \end{aligned} \end{aligned}$$

Using asterisks to denote that $\varvec{\eta }_i$ has been replaced with $\varvec{\eta }_i^*$, we also get the following for the derivative of the second term within the summation of Eq. 23,

29 $$\begin{aligned} \begin{aligned} \frac{d \mathrm {H}_{ikl}(\varvec{\eta }_i^*)}{d \theta _m} = - \frac{1}{2} \sum _{j=1}^{n_{i}} \Bigg (&\frac{d {\mathbf{a}}^{*}_l}{d \theta _m} \, {\mathbf{B}}^{*} \, {\mathbf{a}}^{*T}_k + {\mathbf{a}}^{*}_l \, \frac{d {\mathbf{B}}^{*}}{d \theta _m} \, {\mathbf{a}}^{*T}_k + {\mathbf{a}}^{*}_l \, {\mathbf{B}}^{*} \, \frac{d {\mathbf{a}}^{*T}_k}{d \theta _m}\\&+ {{\mathrm{tr}}}\left[ - \frac{d {\mathbf{c}}^{*}_l}{d \theta _m} \, {\mathbf{c}}^{*}_k - {\mathbf{c}}^{*}_l \, \frac{d {\mathbf{c}}^{*}_k}{d \theta _m} \right] \Bigg ) - \frac{d \mathrm {\Omega }_{kl}^{-1}}{d \theta _m}, \end{aligned} \end{aligned}$$

where

30 $$\begin{aligned} \frac{d {\mathbf{a}}^{*}_k}{d \theta _m}= & {} \frac{d}{d \theta _m} \left( \frac{d \varvec{\epsilon }_{ij}^{T}}{d \eta _{ik}} \right) ^* - \frac{\varvec{\epsilon }_{ij}^{*T}}{d \theta _m} {\mathbf{R}}_{ij}^{*-1} \left( \frac{d {\mathbf{R}}_{ij}}{d \eta _{ik}} \right) ^*\nonumber \\&\quad + \varvec{\epsilon }_{ij}^{*T} {\mathbf{R}}_{ij}^{*-1} \frac{d {\mathbf{R}}^{*}_{ij}}{d \theta _m} {\mathbf{R}}_{ij}^{*-1} \left( \frac{d {\mathbf{R}}_{ij}}{d \eta _{ik}} \right) ^*\nonumber \\&\quad - \varvec{\epsilon }_{ij}^{*T} {\mathbf{R}}_{ij}^{*-1} \frac{d}{d \theta _m} \left( \frac{d {\mathbf{R}}_{ij}}{d \eta _{ik}} \right) ^*,\end{aligned}$$

31 $$\begin{aligned} \frac{d {\mathbf{B}}^{*}}{d \theta _m}= & {} - 2 {\mathbf{R}}_{ij}^{*-1} \frac{d {\mathbf{R}}^{*}_{ij}}{d \theta _m} {\mathbf{R}}_{ij}^{*-1}, \end{aligned}$$

and

32 $$\begin{aligned} \frac{d {\mathbf{c}}^{*}_k}{d \theta _m} = - {\mathbf{R}}_{ij}^{*-1} \frac{d {\mathbf{R}}^{*}_{ij}}{d \theta _m} {\mathbf{R}}_{ij}^{*-1} \left( \frac{d {\mathbf{R}}_{ij}}{d \eta _{ik}} \right) ^* + {\mathbf{R}}_{ij}^{*-1} \frac{d}{d \theta _m} \left( \frac{d {\mathbf{R}}_{ij}}{d \eta _{ik}} \right) ^*. \end{aligned}$$

We now continue to expand the terms in Eqs. 28–32 containing derivatives with respect to $\theta _m$. The terms $d \varvec{{\Omega }}/d \theta _m$ and $d \mathrm {\Omega }_{kl}^{-1}/d \theta _m$ are obtainable by straightforward differentiation. Noting that the terms $\varvec{\epsilon }^*_{ij}$, $(d \varvec{\epsilon }_{ij} / d \eta _{ik})^*$, ${\mathbf{R}}^*_{ij}$, and $(d {\mathbf{R}}_{ij} / d \eta _{ik})^*$, have indirect and/or direct dependence on $\varvec{\theta }$ and $\varvec{\eta }_i^*$, we apply the results from Eq. 26 and expand the remaining derivatives. First,

33 $$\begin{aligned} \frac{d \varvec{\epsilon }^*_{ij}}{d \theta _m} = \left. \frac{d \varvec{\epsilon }_{ij}}{d \theta _m} \right| _{\varvec{\eta }_i =\varvec{\eta }_i^*(\varvec{\theta })} \,+\,              \left.  \frac{d \varvec{\epsilon }_{ij}}{d \varvec{\eta }_i} \right| _{\varvec{\eta }_i = \varvec{\eta }_i^*(\varvec{\theta })} \frac{d \varvec{\eta }_i^*}{d \theta _m}. \end{aligned}$$

Here, $d \varvec{\epsilon }_{ij} / d \varvec{\eta }_i$ was determined previously in Eq. 19, and the derivative in the first term is given by

34 $$\begin{aligned} \frac{d \varvec{\epsilon }_{ij}}{d \theta _m} = \frac{d ({\mathbf{d}}_{ij} - \hat {\mathbf{y}}_{ij})}{d \theta _m} = - \left( \frac{\partial {\mathbf{h}}}{\partial \theta _m} + \frac{\partial {\mathbf{h}}}{\partial {\mathbf{x}}_{ij}} \frac{d {\mathbf{x}}_{ij}}{d \theta _m} \right) . \end{aligned}$$

The sensitivity of the random effect optimum with respect to the fixed effect parameters, $d \varvec{\eta }_i^* / d \varvec{\theta }$, must also be determined, which we will return to later. Then,

35 $$\begin{aligned} \frac{d {\mathbf{R}}^*_{ij}}{d \theta _m} = \left. \frac{d {\mathbf{R}}_{ij}}{d \theta _m} \right| _{\varvec{\eta }_i=\varvec{\eta }_i^*(\varvec{\theta })} +  \left.  \frac{d {\mathbf{R}}_{ij}}{d \varvec{\eta }_i} \right| _{\varvec{\eta }_i=\varvec{\eta }_i^*(\varvec{\theta })} \frac{d \varvec{\eta }_i^*}{d \theta _m}, \end{aligned}$$

where $d {\mathbf{R}}_{ij} / d \varvec{\eta }_i$ was determined in Eq. 20, and

36 $$\begin{aligned} \frac{d {\mathbf{R}}_{ij}}{d \theta _m} = \frac{\partial {\mathbf{R}}_{ij}}{\partial \theta _m} + \frac{\partial {\mathbf{R}}_{ij}}{\partial {\mathbf{x}}_{ij}} \frac{d {\mathbf{x}}_{ij}}{d \theta _m}. \end{aligned}$$

Next,

37 $$\begin{aligned} \begin{aligned}&\frac{d}{d \theta _m} \left( \left. \frac{d \varvec{\epsilon }_{ij}}{d \eta _{ik}} \right| _{\varvec{\eta }_i=\varvec{\eta }_i^*(\varvec{\theta })} \right) \\&\quad = \left. \left( \frac{d}{d \theta _m} \left( \frac{d \varvec{\epsilon }_{ij}}{d \eta _{ik}} \right) \right) \right| _{\varvec{\eta }_i=\varvec{\eta }_i^*(\varvec{\theta })} + \left. \left( \frac{d}{d \varvec{\eta }} \left( \frac{d \varvec{\epsilon }_{ij}}{d \eta _{ik}} \right) \right) \right| _{\varvec{\eta }_i=\varvec{\eta }_i^*(\varvec{\theta })} \frac{d \varvec{\eta }_i^*}{d \theta _m} \\&\quad = \left. \left( \frac{d}{d \theta _m} \left( \frac{d \varvec{\epsilon }_{ij}}{d \eta _{ik}} \right) \right) \right| _{\varvec{\eta }_i=\varvec{\eta }_i^*(\varvec{\theta })} + \sum _l \left. \left( \frac{d}{d \eta _{il}} \left( \frac{d \varvec{\epsilon }_{ij}}{d \eta _{ik}} \right) \right) \right| _{\varvec{\eta }_i=\varvec{\eta }_i^*(\varvec{\theta })} \frac{d \eta _{il}^*}{d \theta _m} \\&\quad = - \Bigg ( \frac{\partial ^2 {\mathbf{h}}}{\partial \eta _{ik} \partial \theta _m} + \frac{\partial ^2 {\mathbf{h}}}{\partial \eta _{ik} \partial {\mathbf{x}}_{ij}} \frac{d {\mathbf{x}}_{ij}}{d \theta _m} + \left( \frac{\partial ^2 {\mathbf{h}}}{\partial {\mathbf{x}}_{ij} \partial \theta _m} + \frac{\partial ^2 {\mathbf{h}}}{\partial {\mathbf{x}}_{ij}^2} \frac{d {\mathbf{x}}_{ij}}{d \theta _m} \right) \frac{d {\mathbf{x}}_{ij}}{d \eta _{ik}}\\&\qquad \left. + \frac{\partial {\mathbf{h}}}{\partial {\mathbf{x}}_{ij}} \frac{d^2 {\mathbf{x}}_{ij}}{d \eta _{ik} d \theta _m} \Bigg ) \right| _{\varvec{\eta }_i=\varvec{\eta }_i^*(\varvec{\theta })} - \sum _l \Bigg ( \frac{\partial ^2 {\mathbf{h}}}{\partial \eta _{ik} \partial \eta _{il}} + \frac{\partial ^2 {\mathbf{h}}}{\partial \eta _{ik} \partial {\mathbf{x}}_{ij}} \frac{d {\mathbf{x}}_{ij}}{d \eta _{il}}\\&\qquad + \left( \frac{\partial ^2 {\mathbf{h}}}{\partial {\mathbf{x}}_{ij} \partial \eta _{il}} + \frac{\partial ^2 {\mathbf{h}}}{\partial {\mathbf{x}}_{ij}^2} \frac{d {\mathbf{x}}_{ij}}{d \eta _{il}} \right) \frac{d {\mathbf{x}}_{ij}}{d \eta _{ik}} \left. + \frac{\partial {\mathbf{h}}}{\partial {\mathbf{x}}_{ij}} \frac{d^2 {\mathbf{x}}_{ij}}{d \eta _{ik} d \eta _{il}} \Bigg ) \right| _{\varvec{\eta }_i=\varvec{\eta }_i^*(\varvec{\theta })} \frac{d \eta _{il}^*}{d \theta _m}, \end{aligned} \end{aligned}$$

where we after the third equality have used the results from Eq. 19. The derivative of $(d {\mathbf{R}}_{ij} / d \eta _{ik})^*$ with respect to $\theta _m$ is done in a highly similar way and is left to the reader as an exercise.

In the above expressions, derivatives of ${\mathbf{h}}$ and ${\mathbf{R}}_{ij}$ are obtained by direct differentiation. The derivatives of the state variables are determined by the previously derived sensitivity equation in Eq. 21 and by the additional sensitivity equations

38 $$\begin{aligned} \frac{d}{d t} \left( \frac{d {\mathbf{x}}_{i}}{d \theta _m} \right)= & {} \frac{\partial {\mathbf{f}}}{\partial \theta _m} + \frac{\partial {\mathbf{f}}}{\partial {\mathbf{x}}_{i}} \left( \frac{d {\mathbf{x}}_{i}}{d \theta _m} \right) \nonumber \\ \left( \frac{d {\mathbf{x}}_i}{d \theta _m} \right) (t_0)= & {} \frac{\partial {\mathbf{x}}_{0i}}{\partial \theta _m},\end{aligned}$$

39 $$\begin{aligned} \frac{d}{d t} \left( \frac{d^2 {\mathbf{x}}_{i}}{d \eta _{ik} d \theta _m} \right)= & {} \frac{\partial ^2 {\mathbf{f}}}{\partial \eta _{ik} \partial \theta _m} + \frac{\partial ^2 {\mathbf{f}}}{\partial \eta _{ik} \partial {\mathbf{x}}_{i}} \frac{d {\mathbf{x}}_{i}}{d \theta _m}\nonumber \\&\quad + \left( \frac{\partial ^2 {\mathbf{f}}}{\partial {\mathbf{x}}_{i} \partial \theta _m} + \frac{\partial ^2 {\mathbf{f}}}{\partial ^2 {\mathbf{x}}_{i}} \frac{d {\mathbf{x}}_{i}}{d \theta _m} \right) \left( \frac{d {\mathbf{x}}_{i}}{d \eta _{ik}} \right) + \frac{\partial {\mathbf{f}}}{\partial {\mathbf{x}}_{i}} \left( \frac{d^2 {\mathbf{x}}_{i}}{d \eta _{ik} d \theta _m} \right) \nonumber \\&\quad \left( \frac{d^2 {\mathbf{x}}_i}{d \eta _{ik} d \theta _m} \right) (t_0) = \frac{\partial ^2 {\mathbf{x}}_{0i}}{\partial \eta _{ik} \partial \theta _m}, \end{aligned}$$

and

40 $$\begin{aligned} \begin{aligned} \frac{d}{d t} \left( \frac{d^2 {\mathbf{x}}_{i}}{d \eta _{ik} d \eta _{il}} \right)&= \frac{\partial ^2 {\mathbf{f}}}{\partial \eta _{ik} \partial \eta _{il}} + \frac{\partial ^2 {\mathbf{f}}}{\partial \eta _{ik} \partial {\mathbf{x}}_{i}} \frac{d {\mathbf{x}}_{i}}{d \eta _{il}}\\&\quad + \left( \frac{\partial ^2 {\mathbf{f}}}{\partial {\mathbf{x}}_{i} \partial \eta _{il}} + \frac{\partial ^2 {\mathbf{f}}}{\partial ^2 {\mathbf{x}}_{i}} \frac{d {\mathbf{x}}_{i}}{d \eta _{il}} \right) \left( \frac{d {\mathbf{x}}_{i}}{d \eta _{ik}} \right) + \frac{\partial {\mathbf{f}}}{\partial {\mathbf{x}}_{i}} \left( \frac{d^2 {\mathbf{x}}_{i}}{d \eta _{ik} d \eta _{il}} \right) \\&\qquad \left( \frac{d^2 {\mathbf{x}}_i}{d \eta _{ik} d \eta _{il}} \right) (t_0) = \frac{\partial ^2 {\mathbf{x}}_{0i}}{\partial \eta _{ik} \partial \eta _{il}}. \end{aligned} \end{aligned}$$

As noted previously, all sensitivity equations must be solved simultaneously with the original differential equations for all but trivial models. However, since one or more parameters in the vector $\varvec{\theta }$ may not appear in the differential equation part of the model (such as parameters appearing only in $\varvec{{\Omega }}$), there may be sensitivities which are trivially zero. If there are $p$ non-trivial sensitivities among the parameters in $\varvec{\theta }$, $q$ non-trivial sensitivities among the parameters in $\varvec{\eta }$, and $n$ state variables, the total number of differential equations that has to be solved in order to be able to compute $\log L_F$ and $d \log L_F / d \varvec{\theta }$ for each individual is

41 $$\begin{aligned} n \big (1 + q\big ) \big (1 + p + q/2\big ). \end{aligned}$$

Finally, we need to determine $d \varvec{\eta }_i^* / d \varvec{\theta }$. At the the optimum of each individual joint log-likelihood we have that

42 $$\begin{aligned} \frac{d l_{i}}{d \varvec{\eta }_i}=\varvec{0}, \end{aligned}$$

or put differently,

43 $$\begin{aligned} \left. \frac{d l_{i}}{d \varvec{\eta }_i} \right| _{\varvec{\eta }_i=\varvec{\eta }_i^*(\varvec{\theta })} = \varvec{0}. \end{aligned}$$

This equality holds for any $\varvec{\theta }$, and thus

44 $$\begin{aligned} \frac{d}{d \varvec{\theta }} \left( \left. \frac{d l_{i}}{d \varvec{\eta }_i} \right| _{\varvec{\eta }_i=\varvec{\eta }_i^*(\varvec{\theta })} \right) = \varvec{0}. \end{aligned}$$

Recognizing that $d l_{i}/d \varvec{\eta }_i$ fulfills the requirements of applying the results from Eq. 26, we can write this as

45 $$\begin{aligned} \frac{d}{d \varvec{\theta }} \left( \left. \frac{d l_{i}}{d \varvec{\eta }_i} \right| _{\varvec{\eta }_i=\varvec{\eta }_i^*(\varvec{\theta })} \right) = \left. \frac{d^2 l_{i}}{d \varvec{\eta }_i d \varvec{\theta }} \right| _{\varvec{\eta }_i=\varvec{\eta }_i^*(\varvec{\theta })} + \left. \frac{d^2 l_{i}}{d \varvec{\eta }_i^2} \right| _{\varvec{\eta }_i=\varvec{\eta }_i^*(\varvec{\theta })} \frac{d \varvec{\eta }_i^*}{d \varvec{\theta }} = \varvec{0}. \end{aligned}$$

By rearranging terms and inverting the matrix, we finally get that

46 $$\begin{aligned} \frac{d \varvec{\eta }_i^*}{d \varvec{\theta }} = - \left( \left. \frac{d^2 l_{i}}{d \varvec{\eta }_i^2} \right| _{\varvec{\eta }_i=\varvec{\eta }_i^*(\varvec{\theta })} \right) ^{-1} \left. \frac{d^2 l_{i}}{d \varvec{\eta }_i d \varvec{\theta }} \right| _{\varvec{\eta }_i=\varvec{\eta }_i^*(\varvec{\theta })}. \end{aligned}$$

The second order derivatives of the individual joint log-likelihoods with respect to the random effect parameters were previously derived in Eq. 13. In contrast to the first order approximation of the Hessian used in the approximate population log-likelihood, the second order derivatives of $\varvec{\epsilon }_{ij}$ and ${\mathbf{R}}_{ij}$ are kept. These are obtained by differentiating Eqs. 19 and 20 once more with respect to $\varvec{\eta }_i$ (not shown). This in turn requires the second order sensitivity equations of the state variables with respect to $\varvec{\eta }_i$, which were previously provided in Eq. 40. In addition to second order derivatives of the individual joint log-likelihoods with respect to the random effect parameters, Eq. 46 also requires the second order mixed derivatives, which are given by

47 $$\begin{aligned} \begin{aligned} \frac{d^2 l_{i}}{d \eta _{ik} d \theta _{m}} =&- \frac{1}{2} \sum _{j=1}^{n_{i}} \Bigg ( 2 \frac{d \varvec{\epsilon }_{ij}^T}{d \theta _{m}} {\mathbf{R}}_{ij}^{-1} \frac{d \varvec{\epsilon }_{ij}}{d \eta _{ik}} - 2 \varvec{\epsilon }_{ij}^T {\mathbf{R}}_{ij}^{-1} \frac{d {\mathbf{R}}_{ij}}{d \theta _{m}} {\mathbf{R}}_{ij}^{-1} \frac{d \varvec{\epsilon }_{ij}}{d \eta _{ik}}\\&\quad + 2 \varvec{\epsilon }_{ij}^T {\mathbf{R}}_{ij}^{-1} \frac{d^2 \varvec{\epsilon }_{ij}}{d \eta _{ik} d \theta _{m}} - \varvec{\epsilon }_{ij}^T {\mathbf{R}}_{ij}^{-1} \frac{d^2 {\mathbf{R}}_{ij}}{d \eta _{ik} d \theta _{m}} {\mathbf{R}}_{ij}^{-1} \varvec{\epsilon }_{ij}\\&\quad + 2 \varvec{\epsilon }_{ij}^T {\mathbf{R}}_{ij}^{-1} \frac{d {\mathbf{R}}_{ij}}{d \eta _{ik}} {\mathbf{R}}_{ij}^{-1} \frac{d {\mathbf{R}}_{ij}}{d \theta _{m}} {\mathbf{R}}_{ij}^{-1} \varvec{\epsilon }_{ij} - 2 \varvec{\epsilon }_{ij}^T {\mathbf{R}}_{ij}^{-1} \frac{d {\mathbf{R}}_{ij}}{d \eta _{ik}} {\mathbf{R}}_{ij}^{-1} \frac{d \varvec{\epsilon }_{ij}}{d \theta _{m}}\\&\quad + {{\mathrm{tr}}}\left[ {\mathbf{R}}_{ij}^{-1} \frac{d {\mathbf{R}}_{ij}}{d \theta _{m}} {\mathbf{R}}_{ij}^{-1} \frac{d {\mathbf{R}}_{ij}}{d \eta _{ik}} + {\mathbf{R}}_{ij}^{-1} \frac{d^2 {\mathbf{R}}_{ij}}{d \eta _{ik} d \theta _{m}} \right] \Bigg )\\&- \varvec{\eta }_i^T \varvec{{\Omega }}^{-1} \frac{d \varvec{{\Omega }}}{d \theta _{m}} \varvec{{\Omega }}^{-1} \frac{d \varvec{\eta }_i}{d \eta _{ik}}. \end{aligned} \end{aligned}$$

Here, all terms have previously been introduced except $d^2 \varvec{\epsilon }_{ij} / d \eta _{ik} d \theta _{m}$ and $d^2 {\mathbf{R}}_{ij} / d \eta _{ik} d \theta _{m}$, which are provided within the derivation of Eq. 37 and through a corresponding derivation involving ${\mathbf{R}}_{ij}$.

### Better starting values for optimization of random effect parameters

Computing the approximate population log-likelihood and its gradient with respect to the parameters $\varvec{\theta }$ requires the determination of $\varvec{\eta }_i^*$ for every individual. The first time $\log L_F$ and its gradient are evaluated it is reasonable to initiate the inner level optimizations for $\varvec{\eta }_i^*$ with $\varvec{\eta }_i=\varvec{0}$. However, in the subsequent steps of the optimization with respect to $\varvec{\theta }$, better starting values for $\varvec{\eta }_i$ can be provided. One way of choosing the starting values $\varvec{\eta }_i^0$ for the optimization of $\varvec{\eta }_i$ is to set them equal to the optimized value from the last step of the outer optimization. If we for simplicity of notation from now on suppress the index of $\varvec{\eta }_i$ denoting the individual, $i$, and instead let the the index $s$ denote the step of the outer optimization with respect to $\varvec{\theta }$, this can be expressed as $\varvec{\eta }^0_{s+1}=\varvec{\eta }^*_s$. This will be particularly helpful as the optimization converges and the steps in $\varvec{\theta }$ become smaller. Using $\varvec{\eta }^*$ from the evaluation of $\log L_F$ as starting value is also a good strategy when computing the gradient of $\log L_F$ by a finite difference approximation.

If the sensitivity approach is used for computing the gradient of $\log L_F$, even better starting values of $\varvec{\eta }$ can be provided. This is accomplished by exploiting the fact that the sensitivity $d \varvec{\eta }^* / d \varvec{\theta }$ happens to be part of the gradient calculation. By making a first order Taylor expansion of the implicit function $\varvec{\eta }^*(\varvec{\theta })$, we propose the following update of the starting values of the random effect parameters

48 $$\begin{aligned} \varvec{\eta }^0_{s+1}=\varvec{\eta }^*_s + \frac{d \varvec{\eta }^*_s}{d \varvec{\theta }} (\varvec{\theta }_{s+1}-\varvec{\theta }_{s}). \end{aligned}$$

**Fig. 1:**
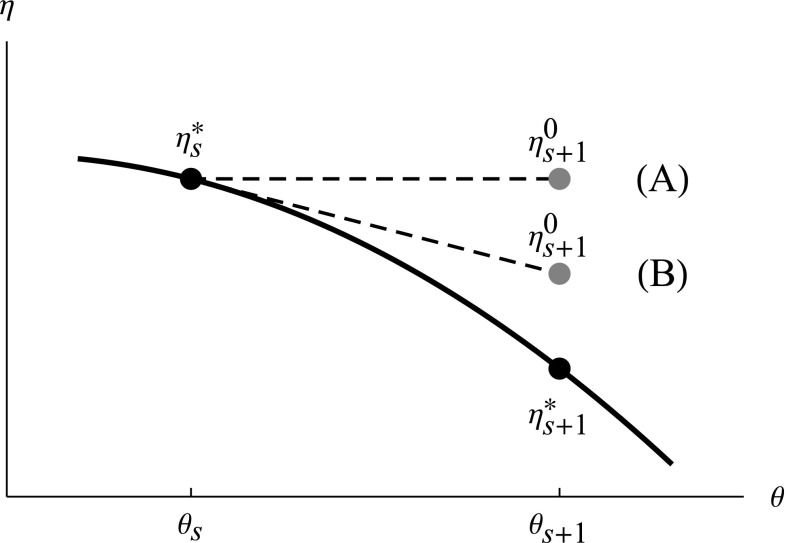
Starting values for finding optimal random parameter values. The hypothetical relationship between a parameter $\theta$ and the optimal value of a random effect parameter $\eta ^*$ is depicted by the *solid curve*, and the optimal values of $\eta$ for two consecutive $\theta$ of the optimization, $\theta _{s}$ and $\theta _{s+1}$, are shown as *black points*. The two approaches for selecting starting values $\eta ^0_{s+1}$ are shown as *dashed lines* and *gray points*, with the label (*A*) for using the previous value and (*B*) for using the gradient based update

The two approaches for choosing $\varvec{\eta }^0_{s+1}$ are illustrated in Fig. 1.

## Results

Based on the theory presented in the previous section, we propose an alternative implementation of the FOCE and FOCEI methods for parameter estimation of NLME models based on differential equations. The steps of this novel approach are outlined in Algorithm 1. The crucial points are the computation of gradients using sensitivity equations, for both the inner and outer problem, and the way that starting values for the inner problem are determined. 
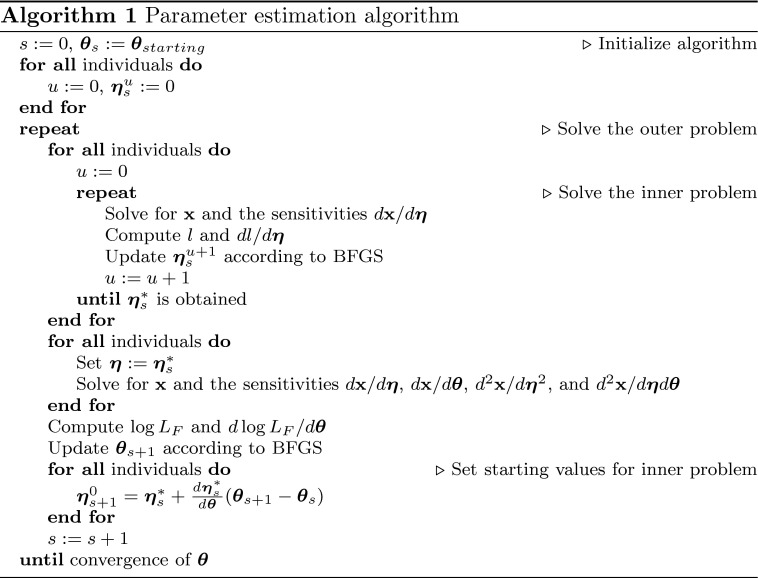


The algorithm was evaluated using a two-compartment model with a capacity-limited elimination. This is a moderately complex pharmacokinetic model that requires the numerical solution of differential equations. All details regarding the model, including model equations, parameters used for simulating data, the starting values for the parameter estimation, and the parameter estimates, can be found in the "Appendix 3" section. A short summary of the model is shown in Table 1. Briefly, four versions of the model (M1-M4) were used. In model M1, some parameters were fixed to the true values, hence excluded from the estimation. Three random effect parameters were introduced but their covariance matrix was limited to a diagonal matrix. Observations were modeled using a normally distributed additive error. All parameters were estimated in model M2, including the full covariance matrix for the random effect parameters. In model M3, an additional random effect parameter was introduced and the full covariance matrix was extended accordingly. The observational model was also altered to include measurements from both compartments, and the error in the measurements from the first compartments was modeled with both an additive and proportional term. Model M4 is the same as M3 but for this model the parameter estimation was performed with FOCEI instead of FOCE.

**Table 1:** Overview of benchmark models showing the method used, the numbers of different types of parameters, and the total number of ordinary differential equations (ODEs) per individual for the inner and outer problem (including the number of sensitivity equations according to Eqs. 22 and 41)

Model	M1	M2	M3	M4
Method	FOCE	FOCE	FOCE	FOCEI
Total number of fixed effect parameters ($\varvec{\theta }$)	6	12	18	18
Parameters in the ODE model	3	5	5	5
Parameters in the observational model	0	1	3	3
Parameters in the random effect covariance matrix	3	6	10	10
Number of random effect parameters ($\varvec{\eta }$)	3	3	4	4
ODEs per individual, inner problem	8	8	10	10
ODEs per individual, outer problem	44	60	80	80

### Improving gradient precision and accuracy

**Fig. 2:**
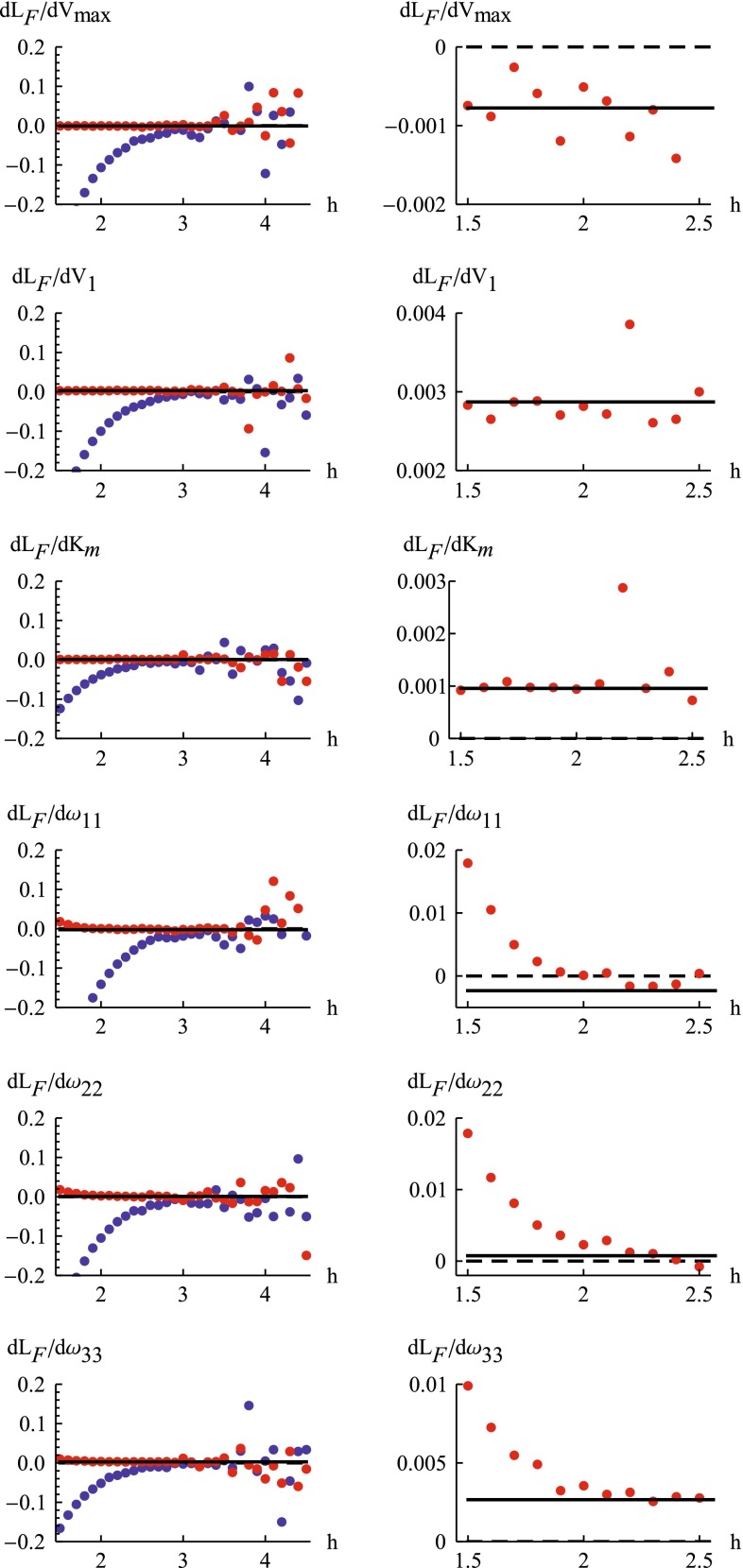
Precision and accuracy of the approximate population log-likelihood gradient. Each *row* displays one element of the gradient, and the *left* and *right* columns show two different levels of magnification, respectively. Evaluations of the derivatives of $\log L_F$ using forward and central differences with different relative step sizes are shown as *blue* and *red dots*, respectively. A single evaluation of the derivatives using the approach based on sensitivity equations is indicated by a *black line*, and the value zero is shown as a *dashed line* for comparison

We compared our proposed method of computing the gradient of the approximate population log-likelihood, $\log L_F$, with respect to $\varvec{\theta }$ to the more straightforward approach of finite difference approximation. Two versions of the finite difference approximations were considered, a forward difference and a central difference. To investigate the precision and accuracy of these approximations, we first determined the estimate of $\varvec{\theta }$ for model M1. We then computed all 6 elements of the gradient at this point in parameter space using different values of the relative step size, $10^{-h}$. The details of the comparison are explained in the methods section. In addition, we computed the gradient using the approach based on sensitivity equations. A comparison of the two approaches is shown in Fig. 2, where each row shows one element of the gradient at two levels of magnification.

The left column of Fig. 2 shows a pattern that appears to be consistent for all parameters; for large $h$, i.e. small step sizes, the result of the finite difference approach is dominated by numerical noise for both forward and central differences. Thus, for this particular model, and for this particular point in parameter space, the finite difference approximations have low precision as $h$ increases beyond 3. For small $h$, i.e. large step sizes, there is a trend of severely decreased accuracy for the forward differences. Looking at the values of the gradient from the approach of sensitivity equations, it is clear that for $h$ around 2 and smaller, forward differences produces values of elements of the gradient that are up to two orders of magnitude larger, and with a wrong sign in four of six cases. The behavior of the central difference approximation for small and intermediate $h$ is best viewed in the right column, where the scales of the axis have been chosen differently. For the three first elements of the gradient, namely the derivatives of $\log L_F$ with respect to $V_{max}$, $V_1$, and $K_m$, the central difference approximation appears to be accurate but, on the scale of the size of the gradient computed according to the sensitivity equation approach, the limits in precision are visible. For the derivatives with respect to the the parameters of $\varvec{{\Omega }}$, $\omega _{11}$, $\omega _{22}$, and $\omega _{33}$, there are obvious issues with both accuracy and precision of the approximation, producing derivatives that are both of wrong size and sign. The fact that the approximation starts to deviate systematically for $h$ less than 2 indicates that in these parameter directions, and on this scale, an expansion of the approximate log-likelihood function has a significant contribution of third order terms and higher, causing a bias in the approximation of the gradient using central differences.

The approach of determining the gradient using sensitivity equations is also subject to numerical errors. By repeated evaluation of the gradient using randomized values for the starting values of the inner optimization problem, we determined the relative standard error. For all 6 parameter directions of the gradients, the relative standard errors were between 0.1 and 1 %. Thus, these numerical errors are so small that they would not even be visible on the scales of Fig. 2.

### Improving computational time

**Fig. 3:**
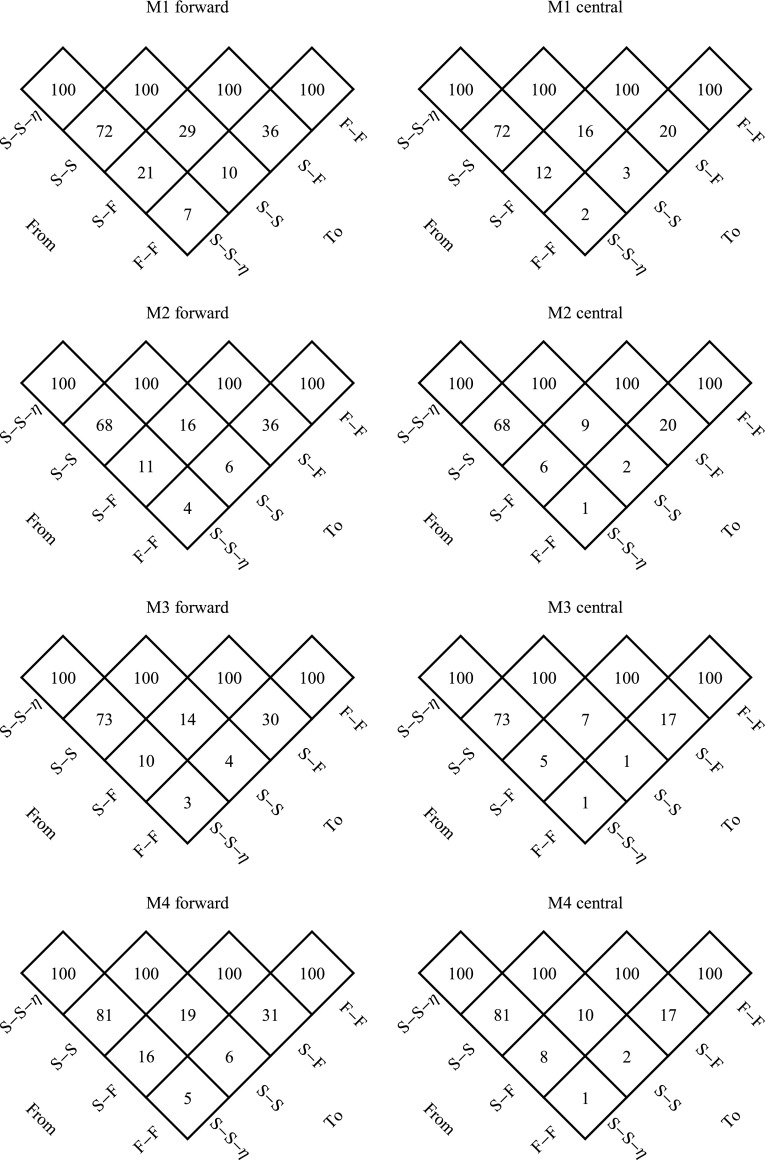
Comparison of relative estimation times. The relative computation times expressed in percentage are shown for going from one scheme for obtaining gradients to another. Results are shown for the model variants M1-M4, using either a forward or central implementation of the finite difference approach. F-F denotes the use of finite differences for both the inner and outer problem, S-F the use of gradients based on sensitivity equations for the inner problem, S-S the use of gradients based on sensitivity equations for both inner and outer problems, and S-S-$\eta$ denotes the additional implementation of the better starting values for the inner problem

We investigated the improved computational times resulting from replacing finite difference approximations of the gradients in the inner and outer problem with gradients computed using sensitivity equations, and from using better starting values for the inner problems. The contribution from each of these three steps, as well as their accumulative effect, are shown in Fig. 3.

For the first step of improvement, using gradients based on sensitivity equations for the inner problem, computational times for models M1 and M2 (with 3 random effect parameters) decreased to almost a third compared to the approximation using forward differences, and to a fifth compared to central differences. The ratio of these two relative decreases is reasonable considering that the forward difference approximation requires 4 function evaluations and the central difference requires 7 evaluations. Model M3 and M4 contain one additional random effect parameter and the gains in speed were slightly larger compared to both variants of the finite difference approximation.

Replacing the finite difference approximation of the gradient in the outer problem with the approach based on sensitivity equations results in further improvement of computational times. As the number of parameters in the outer optimization problem increase from 6 to 18 for the models M1 to M3, the reduction in computational times improves from 29 to 14 % when compared to forward differences, and from 16 to 7 % compared to central differences. Although model M4 is identical to M3, the reduction in computational times are slightly less for this model. This is because M4 uses FOCEI for estimating parameters, which compared to FOCE requires more time for putting together the more complex gradient expressions once the sensitivity equations have been solved. Again it is reasonable to expect a nearly doubled factor of decrease when comparing central and forward finite differences since the former need almost twice as many function evaluations.

The final step of improvement is only applicable when gradients for both the inner and outer problem are computed using the approach based on sensitivity equations. Thus, the distinction between forward and central differences is no longer of importance. The decrease in computational times were around 70 % for models M1 to M3, and somewhat less for model M4, which again benefits less due to its larger overhead of having to compute all interaction terms.

The accumulated effect of all the steps range from a decrease in computational times to 7 % for the least complex model when comparing to forward differences, to the substantial decrease to 1 % for the most complex model when comparing to central differences.

## Discussion

This article has demonstrated a novel approach to the computation of gradients needed for the FOCE and FOCEI approximation of the population likelihood encountered in NLME modeling. We have derived the analytic expressions for the gradients of both the individual and population log-likelihoods as well as the so called sensitivity equations, whose solution is a necessity for evaluating the gradient expressions.

Using sensitivity equations to compute the gradient for the inner problem is quite straightforward. As we understand it, approaches along these lines are in fact used for the inner problem, at least to some extent, in softwares such as NONMEM and Phoenix NLME. For the approximate population log-likelihood on the other hand, the sensitivity approach to gradient computation is complicated by the fact that this function depends on the nested optimization of the individual joint log-likelihoods. In this work we have, to the best of our knowledge, for the first time demonstrated how sensitivity equations can be used for computing the gradient of the FOCE and FOCEI approximations to the population log-likelihood. A key step to obtain this gradient involves the derivative of the optimal random effect parameters with respect to the fixed effect parameters. It was shown that this derivative could be determined given second order sensitivity equations.

Abandoning the finite difference approximation of gradients in favor of the approach of sensitivity equations were shown to have two advantages; gradients could be computed with a higher precision and computational times were substantially reduced. Though, implementation of the presented method is more challenging compared to finite difference FOCE/FOCEI, and the limitations of the Laplacian approximation are still present.

### Increased precision and accuracy of gradients

The optimization of the approximate population log-likelihood $\log L_F$ with respect to $\varvec{\theta }$ would typically be performed with a quasi-Newton method. A straightforward approach to obtaining the gradient needed for such methods is to compute it from a finite difference approximation. However, the finite difference approach may result in issues with both precision and accuracy of the gradient. We demonstrated this for the computation of the gradient in the outer problem, evaluated close to the optimum of $\log L_F$. Although the use of central differences with an appropriate step length could avoid the worst problems, precision and accuracy were still inferior compared to the approach based on sensitivity equations. The potential limitations of combining NLME models based on differential equations with likelihood optimization using gradients computed by finite differences have previously been recognized [3]. The issues with the finite difference approximation depend both on numerical limitations and on the approximation itself. First of all, evaluation of $\log L_F$ can only be done to a certain precision. This is especially evident for models based on differential equations, whose solution involves adaptive schemes for numerical integration. In addition to the numerical precision of functions like $\log$, which is high, the precision of $\log L_F$ depends on the precision of the solutions to the differential equations, and the precision of computing derivatives with respect to $\varvec{\eta }$. The precision of $\log L_F$ also has a strong dependence on the precision of $\varvec{\eta }^*$, which in turn again depends on the solutions of differential equations and, if the inner level optimization problem is performed using a gradient-based method, depends on computing derivatives of the individual joint log-likelihoods with respect to $\varvec{\eta }$. Secondly, taking finite differences of $\log L_F$ will amplify numerical errors, resulting in increasingly poor precision of the gradient as the step size is decreased. On the other hand, taking too long steps will decrease the accuracy of the approximation due to the increasing impact of higher order terms in an expansion of $\log L_F$ (forward differences is only exact up to first order terms, and central differences is only exact up to second order terms). Even if it for a given model in some cases would be possible to customize the step length for the finite difference approximation (which typically would be different in each separate parameter direction) using an analysis like the one performed here, it would be infeasible in practice since such an investigation may take longer time than solving the parameter estimation problem itself. Adding further to the problem, the choice of a suitable step size will most certain be different depending on the point in parameter space, thus constantly requiring a reevaluation of the step size.

There are several advantages of being able to compute gradients with an improved precision and accuracy (i) Parameter estimates can be computed with higher precision, or alternatively, the same precision can be obtained but with shorter run times since we may afford to reduce the precision of the inner problem while still maintaining a similar precision in the outer problem [11]. (ii) Premature termination and convergence problems of the parameter estimation algorithm can be avoided or at least reduced [8, 24]. (iii) May enable the calculation of standard errors of the parameter estimates in cases where this was not possible due to the numerical issues of the finite difference approach [7]. However, we want to point out that for many points in the parameter space the limited precision and accuracy of the finite difference approach may not be crucial for the progression of the optimization as long as the approximation of the gradient results in a true ascent direction of the function being maximized.

### Decreased computational times

The relative decrease in computational times were investigated for the successive application of three specific steps toward improvement, namely (i) Gradients based on sensitivity equations in the inner problem, (ii) Gradients based on sensitivity equations in the outer problem, and (iii) Better starting values for the inner problem. In all cases of applying the two first steps, we found that the decrease in computational times were substantially larger when comparing to central differences instead of forward differences. This was anticipated since central differences requires almost twice as many function evaluations as forward differences. Moreover, for both the inner and outer levels of optimization, the gains in computational times tended to be larger for models with higher number of parameters. For instance, the run time improvements of providing gradients from sensitivity equations in the outer problem were more than doubled for model M3 with 18 parameters compared to model M1 with 6 parameters. It was also observed that the improvement factor in the outer optimization was slightly lower for FOCEI compared to FOCE. Although the number of ODEs to be solved in both the inner and outer problem is the same, this was expected considering that the FOCEI method is based on more extensive expressions for both the likelihood and its gradient.

There are two main reasons why the approaches based on sensitivity equations should be faster. First of all, the right hand side of the sensitivity equations has lots of common subexpressions both with other sensitivity equations and with the original system of differential equations. Thus, the cost of evaluating the right hand side for the combined system of the original differential equations and the sensitivity equations can be surprisingly small. Furthermore, since the sensitivity equations are linear in the sensitivity state variables, there is typically little extra effort needed in the adaptive time stepping of the differential equations solver for accommodating these additional equations. For the inner problem this means that it is faster to solve the combined system, yielding in total $n (1+q)$ differential equations, rather than having to solve the $n$ original differential equations $1+q$ times, which would have been the case using forward finite differences. Secondly, the use of sensitivity equations in the outer level optimization avoids the repeated need of having to solve the inner problems for perturbed values of the outer parameters. The exact improvement made at this step depends on several factors of which perhaps the most important one is the desired precision (and hence the number of iterations required) of the inner optimizations needed for every parameter perturbation of a finite difference approximation (had this alternative been used instead).

We furthermore note that the computation of gradients based on sensitivity equations is highly amenable to parallelization, something which may be exploited to speed up computations considerably. The potential gains of doing this are expected to be similar to those of parallelizing the computation of the population log-likelihood itself [11].

In addition to the reduced computational times coming from the two steps of improved gradient computations, a third level of speed up was obtained by choosing more informed starting values for the inner problem. Although this improvement was not as substantial as the others, the gains from this step may be quite dependent on the starting values of the outer optimization problem. As the outer level optimization converges, the steps in $\varvec{\theta }$ become successively smaller, which in turn means that the linear approximation of $\varvec{\eta }^*(\varvec{\theta })$ becomes better. Thus, the overall improvement in computational time will depend on how much of the optimization that was spent in these “later stages” of convergence. This means that it is likely that the relative improvement will be larger if the optimization had been started closer to the optimum.

Setting the results of Fig. 3 in relation to commercial softwares for NLME parameter estimation, we would like to comment on a mixed analytical/finite difference approach to the differentiation of the FOCE likelihood with respect to the parameters of the random effect covariance matrix $\varvec{{\Omega }}$, which is used as default by NONMEM (when the SLOW option is not selected). Since these parameters do not normally directly influence neither the residuals, nor the residual covariance matrix, their part of the likelihood gradient is less complicated compared to other parameters. As shown by the theory in this paper, their part of the gradient may be computed using only second order $\varvec{\eta }$ sensitivities (Eq. 40), not requiring first order $\varvec{\theta }$ or second order mixed sensitivities (Eqs. 38 and 39, respectively). Although NONMEM FOCE does not use second order $\varvec{\eta }$ sensitivities, it still utilizes this technique by performing a central finite difference evaluation on the first order $\varvec{\eta }$ sensitivities. While this is slower than performing completely analytical second derivatives, along with some erosion of precision, it is certainly faster than the SLOW FOCE method, which must perform the inner problem re-optimizations at each outer level perturbation of the $\varvec{{\Omega }}$-parameters. The derivatives of the likelihood with respect to the remaining parameters are still obtained from finite differences.

The degree of improvement of speed for the S-S approach compared to an approach that is mixing finite differences and analytical methods at the outer level, i.e, an S-F/S approach, may therefore be less substantial than what can be achieved for going from S-F to S-S. Under the realistic assumption that all perturbed evaluations of $\log L_F$ are equally costly, and further assuming that the $\varvec{{\Omega }}$-part of the gradient can be obtained at a computationally insignificant cost (ignoring the relatively few extra evaluations needed for the central finite difference of the first order $\varvec{\eta }$ sensitivities), the reference time of 100 % for going from forward differences S-F to S-S in Fig. 3 would change to $((1+P_{\theta }-P_{{\Omega }})/(1+P_{\theta }))100$ % if instead going from S-F/S to S-S, where $P_{\theta }$ is the total number of parameters and $P_{{\Omega }}$ is the number of $\varvec{{\Omega }}$-parameters. The reference time for going from central differences S-F to S-S would for S-F/S to S-S similarly change to $((1+2P_{\theta }-2P_{{\Omega }})/(1+2P_{\theta }))100$ %. For model M1 this would mean that the improvements to 29 and 16, for forward and central differences, respectively, should be compared to the S-F/S references of 57 and 54, rather than to 100, and for model M3 the improvements to 14 and 7 should be compared to 47 and 46. In general, one would expect the advantage of the S-S approach to decrease as the fraction of $\varvec{{\Omega }}$-parameters with respect to the total number of parameters increases, e.g., for problems with many random effect parameters when estimating the full random effect covariance matrix. It must however be emphasized that this is a mixed analytical/finite difference approach, and may as such have lower precision and accuracy compared to the S-S approach. Moreover, the remaining part of the gradient will still be completely derived from finite differences, and is expected to have the same comparable quality to the S-S approach as demonstrated in the results section.

Extending the line of thought, one could also consider a hybrid between the above S-F/S approach and the S-S approach, where the derivatives of $\log L_F$ with respect to the $\varvec{{\Omega }}$-parameters are computed according to the exact approach presented in this work but where the derivatives for the remaining parameters of the outer level problem are obtained from a finite difference approach. This would indeed require the second order sensitivity equations with respect to $\varvec{\eta }$, but not the first order $\varvec{\theta }$ or the mixed second order sensitivity equations. The accuracy and precision would still be lower for the part of the gradient obtained from finite differences but the elements corresponding to the parameters of $\varvec{{\Omega }}$ would be of the same quality as the S-S approach, i.e., without approximations.

### Challenges and limitations

Moving from a convenient proof-of-concept environment such as Mathematica, in which the proposed method currently is implemented, to a more stand-alone environment of a commercial software may present various challenges. One of the most obvious challenges is the integration of functionality for performing symbolic differentiation. This is essential since the sensitivity equations, i.e., the differential equations in Eqs. 21, 38, 39, and 40, are model specific and have to be derived for every new model, in order to apply the results of this paper. It also applies to the derivatives of ${\mathbf{h}}$, ${\mathbf{R}}_{ij}$, and $\varvec{{\Omega }}$, which too are model specific. Since differential equation models may be quite complex, and because second order derivatives are needed, it is not realistic to perform these derivations manually, and a tool that can perform symbolic differentiation will be required. To this end, one may consider to look at free symbolic packages such a SymPy [23]. The use of tools for symbolic analysis may furthermore be crucial to exploit the existence of common subexpressions, e.g., in the right hand sides of the sensitivity equations.

An alternative approach, which does not require symbolic differentiation, would be to use so called automatic differentiation (AD) [19]. The idea of AD is that every mathematical function that can be written as a computer program can be differentiated by applying the chain rule of differentiation, leading to the differentiation of every elementary operation of that computer program. Even though AD in principle could be applied directly to the approximate population likelihood, whose gradient we wish to compute, this would in practice be infeasible as this function is based on the execution of both optimization routines and adaptive numerical integration of differential equations. If used, AD would therefore not be applied to the population likelihood, but to the right hand sides of the model differential equations, and to the other model objects requiring differentiation. The parameter estimation would thus still proceed according to the steps laid out in Algorithm 1, but with symbolic differentiation replaced with AD. Following such an approach, the precision and accuracy of the gradients are not expected to differ, but it would have to be investigated how AD performs in terms of computational times. With a so called reverse mode AD it may actually be possible to improve run times even further compared to the current results.

Even if tools for differentiation can be provided for a stand-alone implementation, estimation methods which involve the direct differentiation of model state variables, etc., may experience limitations when considering other types of mathematical formalisms, such as models based on stochastic differential equations or hidden Markov models, since the required derivatives may be challenging to obtain. The method of computing gradients based on finite differences, on the other hand, do not care about the details of how a model is evaluated and has no limitations in this sense.

Finally, it should also be mentioned that although the approach for gradient computations presented here may improve the performance of FOCE and FOCEI, the fundamental limitations of the Laplacian approximation as such still remains. Being only an approximation to the population likelihood, this class of methods do not guarantee the desirable statistical properties of a true maximum likelihood estimate. In this respect the new generation of estimation methods which are based on Monte Carlo expectation maximization methods, such as stochastic approximation expectation maximization and importance sampling, are superior to the classical ones since the parameter estimates and their confidence intervals, etc., are not biased by likelihood approximations. However, FOCE and FOCEI will likely be important complementary methods for a long time still, and improving their efficiency is therefore nonetheless relevant.

### Possible extensions

The approach of computing gradients using sensitivity equations presented here could be modified for other variants of the population likelihood based on the Laplacian approximation. For instance, with some alterations it could be applied to the first order (FO) approximation of the population likelihood. Since the FO method does not rely on conditioning with respect to the optimal random effect parameters, the use of an approach based on sensitivity equations would be less complicated but at the same time also less rewarding. Gradients based on the approach of sensitivity equations could with some adjustments also be derived for the Laplace method. This would however require third order sensitivity equations but may be worthwhile since the potential gains should be at least as substantial as for FOCE and FOCEI. Because the theory presented in this article is derived for the FOCEI approximation, it accounts for the dependence of residual errors on the random effect parameters. This means that the gradient expressions stated here are suitable for prediction error-type NLME models, including models based on stochastic differential equations (see for instance [6, 14, 18]), since these typically display an interaction between residuals and random effects. The first step towards this end has in fact already been taken through the successful application of sensitivity equations for computing gradients in stochastic differential equation models on the single-subject level [16]. Furthermore, gradient computations based on sensitivity equations may be useful for the problem of optimal experimental design [1, 17].

### Conclusions

The presented approach of computing gradients for both the individual- and population-level log-likelihoods of the FOCE and FOCEI approximations leads to more robust gradients and decreased computational times. We therefore suggest that future implementations of these conditional estimation methods should include the approach based on sensitivity equations for computing the gradients. We eagerly await the further development of the proposed approach from the prototyped version used in the present study to its implementation in publicly or commercially available software packages.

## Methods

The NLME parameter estimation algorithm investigated in this study was implemented in Mathematica 9. An executable version of the code, and the data sets used within this study, may be received from the authors upon request.

### Comparison of performance

The performance of a computer program for parameter estimation in NLME models depends on several factors, such as the particular NLME model, the experimental data, how the estimation problem is formulated and possibly approximated, the choice and settings of the optimization method (including sub-methods such as line-searches, etc.), starting values of parameters, the differential equation solver used, the design of convergence criteria, etc. This paper is investigating the advantages of providing gradients by means of sensitivity equations for the FOCE or FOCEI approximation of the population likelihood. However, this paper is not claiming to address all the other factors that will impact on the parameter estimation. Comparing measures such as absolute run-times of our implementation with commercial software like NONMEM may therefore be misleading with respect to the advantages of gradient calculations. To avoid this the comparison is designed to look only at the improvements made by abandoning the finite difference approximation in our own implementation.

#### Comparison of precision and accuracy

The comparison of precision and accuracy was performed in the following way. At the optimal values of $\varvec{\theta }$ (found from the comparison of computational times), the elements of the gradient of the approximate log-likelihood function were approximated with finite differences, using a relative step size, according either to a forward difference

49 $$\begin{aligned} \frac{\log L_F \big (\theta _m (1+10^{-h})\big ) - \log L_F(\theta _m)}{\theta _m 10^{-h}}, \end{aligned}$$

or a central difference,

50 $$\begin{aligned} \frac{\log L_F\big (\theta _m (1+10^{-h})\big ) - \log L_F\big (\theta _m (1-10^{-h})\big )}{2 \theta _m 10^{-h}}. \end{aligned}$$

For these function evaluations, the inner problem was solved to a precision of 4 digits (using the gradients from the approach of sensitivity equations). Furthermore, for forward differences the value of $\log L_F$ was recalculated for every $h$ using randomized starting values for the inner problems. This was done to avoid correlations between differences with different step size that may otherwise have resulted from a single realization of the numerical error of $\log L_F$.

The approach of determining gradients using sensitivity equations does not involve any approximations, and is therefor expected to be correct on average. Its precision was assessed by computing the gradient 500 times using randomized starting values for the inner problems. For these gradient evaluations, the inner problem was solved to a precision of 4 digits.

#### Comparison of computational times

The comparison of computational times was done in the following way. Both the inner and outer problem were solved using gradients based on sensitivity equations, as outlined in the theory section. The inner problem was solved to a precision of 4 digits, and the outer to a precision of 3 digits. The comparison to finite differences was done by simultaneously clocking the time of computing gradients by a finite difference approximation but proceeding with the optimizations according to values of the gradient from the sensitivity approach. The reason for doing this is that the number of iterations, and the properties of every iteration (such as stiffness of the model equation with that certain set of parameters), for solving both the inner and outer problem might be affected by the choice of method for computing the gradients. Even small numerical differences in the results of the two methods may cause the paths taken in the parameter space to diverge substantially over the course of the optimizations, potentially making the comparison unfair. In this way we isolate the comparison to the actual computational times for the different methods of obtaining the gradients. Since the methods based on sensitivity equations were shown to have a higher precision in the evaluation of gradients, there may be additional gains in computational times to be made from traversing the parameter space based on more exact gradients. However, quantifying this type of contribution may require averaging over a large number of models and parameter starting values and was not considered. Thus, our implementation of the comparison focuses on the direct improvements in computational times and will therefore be a conservative measure of the gains in speed.

To make a fair implementation of timing the finite differences approach the following starting values of the random effect parameters for the inner problem were used. When evaluating the approximate population log-likelihood at the unperturbed parameter values of the outer problem, the starting values for the parameters of the inner problem were set to the optimum from the previous outer evaluation, i.e., according to approach A in Fig. 1. For evaluating the approximate population log-likelihood at the perturbed parameter values of the outer problem, the starting values for the parameters of the inner problem were set to the optimum obtained for the unperturbed outer problem parameters. The relative size of each perturbation of the parameters in $\varvec{\theta }$ was $10^{-2}$.

Compared to the finite difference approaches, using sensitivity equations had an overhead of evaluating the quite substantial mathematical expressions for the gradients once the differential equations are integrated, something which was carefully included in the comparison of computational times.

### Optimization algorithm

Both the inner and outer optimization problems were solved using the BFGS method [20].

### Derivation of sensitivity equations

Given an NLME differential equation model, the corresponding sensitivity equations were derived by symbolic differentiation in Mathematica.

## Appendix 1: Matrix calculus

The default representation of a vector is a column vector,

51 $$ {\mathbf{y}} = \left( {\begin{array}{*{20}c}    {{{y}}_{1} }  \\    {{{y}}_{2} }  \\     \vdots   \\    {{{y}}_{m} }  \\   \end{array} } \right) .$$

The derivatives of vectors and matrices by scalars are defined as element-wise derivatives, according to

52 $$\frac{{d{\mathbf{y}}}}{{dx}} = \left( {\begin{array}{*{20}c}    {\frac{{dy_{1} }}{{dx}}}  \\    {\frac{{dy_{2} }}{{dx}}}  \\     \vdots   \\    {\frac{{dy_{{m}} }}{{dx}}}  \\   \end{array} } \right), $$

and

53 $$  \frac{{d{\mathbf{A}}}}{{dx}} = \left( {\begin{array}{*{20}c}    {\frac{{da_{{11}} }}{{dx}}} & {\frac{{da_{{12}} }}{{dx}}} & { \cdots } & {\frac{{da_{{1{n}}} }}{{dx}}}  \\    {\frac{{da_{{21}} }}{{dx}}} & {\frac{{da_{{22}} }}{{dx}}} & {\cdots } & {\frac{{da_{{2{n}}} }}{{dx}}}  \\     \vdots  & {\vdots } & {\ddots } & {\vdots }  \\    {\frac{{da_{{{m}1}} }}{{dx}}} & {\frac{{da_{{{m}2}} }}{{dx}}} & {\cdots } & {\frac{{da_{{{mn}}} }}{{dx}}}  \\   \end{array} } \right),  $$

respectively. The derivative of scalar by vector is given by

54 $$ \frac{{dy}}{{d{\mathbf{x}}}} = \left( {\begin{array}{*{20}c}    {\frac{{dy}}{{dx_{1} }}} & {\frac{{dy}}{{dx_{2} }}} &  \cdots  & {\frac{{dy}}{{dx_{m} }}}  \\   \end{array} } \right), $$

the derivative of vector by vector is given by

55 $$  \frac{{d{\mathbf{y}}}}{{d{\mathbf{x}}}} = \left( {\begin{array}{*{20}c}    {\frac{{dy_{1} }}{{dx_{1} }}} & {\frac{{dy_{1} }}{{dx_{2} }}} &  \cdots  & {\frac{{dy_{1} }}{{dx_{n} }}}  \\    {\frac{{dy_{2} }}{{dx_{1} }}} & {\frac{{dy_{2} }}{{dx_{2} }}} &  \cdots  & {\frac{{dy_{2} }}{{dx_{n} }}}  \\     \vdots  &  \vdots  &  \ddots  &  \vdots   \\    {\frac{{dy_{m} }}{{dx_{1} }}} & {\frac{{dy_{m} }}{{dx_{2} }}} &  \cdots  & {\frac{{dy_{m} }}{{dx_{n} }}}  \\   \end{array} } \right),  $$

and the derivative of row-vector by vector is given by

56 $$  \frac{{d{\mathbf{y}}^{T} }}{{d{\mathbf{x}}}} = \left( {\begin{array}{*{20}c}    {\frac{{dy_{1} }}{{dx_{1} }}} & {\frac{{dy_{2} }}{{dx_{1} }}} &  \cdots  & {\frac{{dy_{m} }}{{dx_{1} }}}  \\    {\frac{{dy_{1} }}{{dx_{2} }}} & {\frac{{dy_{2} }}{{dx_{2} }}} &  \cdots  & {\frac{{dy_{m} }}{{dx_{2} }}}  \\     \vdots  &  \vdots  &  \ddots  &  \vdots   \\    {\frac{{dy_{1} }}{{dx_{m} }}} & {\frac{{dy_{2} }}{{dx_{m} }}} &  \cdots  & {\frac{{dy_{m} }}{{dx_{n} }}}  \\   \end{array} } \right). $$

The derivative of a quadratic form is obtained in the following way. Let $y = {\mathbf{b}}^T {\mathbf{A}} {\mathbf{b}}$, where ${\mathbf{A}}$ is a square matrix and ${\mathbf{b}}$ a suitable vector. If ${\mathbf{A}}$ is symmetric then

57 $$\begin{aligned} \begin{aligned} \frac{dy}{dx}&= \frac{d{\mathbf{b}}^T}{dx} {\mathbf{A}} {\mathbf{b}} + {\mathbf{b}}^T \frac{d{\mathbf{A}}}{dx} {\mathbf{b}} + {\mathbf{b}}^T {\mathbf{A}} \frac{d {\mathbf{b}}}{dx}\\&= {\mathbf{b}}^T {\mathbf{A}}^T \frac{d{\mathbf{b}}}{dx} + {\mathbf{b}}^T \frac{d{\mathbf{A}}}{dx} {\mathbf{b}} + {\mathbf{b}}^T {\mathbf{A}} \frac{d {\mathbf{b}}}{dx}\\&= 2 {\mathbf{b}}^T {\mathbf{A}} \frac{d{\mathbf{b}}}{dx} + {\mathbf{b}}^T \frac{d{\mathbf{A}}}{dx} {\mathbf{b}}. \end{aligned} \end{aligned}$$

The derivative of an inverse matrix is found by noting that

58 $$\begin{aligned} \frac{d{\mathbf{A}}^{-1}}{dx} = \frac{d \left( {\mathbf{A}}^{-1} {\mathbf{A}} {\mathbf{A}}^{-1} \right) }{dx} = \frac{d{\mathbf{A}}^{-1}}{dx} {\mathbf{A}} {\mathbf{A}}^{-1} + {\mathbf{A}}^{-1} \frac{d{\mathbf{A}}}{dx} {\mathbf{A}}^{-1} + {\mathbf{A}} {\mathbf{A}}^{-1} \frac{d{\mathbf{A}}^{-1}}{dx}, \end{aligned}$$

and thus that

59 $$\begin{aligned} \frac{d{\mathbf{A}}^{-1}}{dx} = - {\mathbf{A}}^{-1} \frac{d{\mathbf{A}}}{dx} {\mathbf{A}}^{-1}. \end{aligned}$$

The derivative of the logarithm of the determinant of a covariance matrix is given by the following expression. If ${\mathbf{A}}$ is a real-valued, symmetric, positive-definite matrix, then

60 $$\begin{aligned} \frac{d}{dx} \log |{\mathbf{A}}| = {{\mathrm{tr}}}\left[ {\mathbf{A}}^{-1} \frac{d{\mathbf{A}}}{dx} \right] . \end{aligned}$$

This can be seen by first writing ${\mathbf{A}}$ as ${\mathbf{A}} = {\mathbf{Q}} \varvec{{\Lambda }} {\mathbf{Q}}^{-1}$, where $\varvec{{\Lambda }}$ is a diagonal matrix. Now, the left-hand side of Eq. 60 becomes

61 $$\begin{aligned} \begin{aligned} \frac{d}{dx} \log |{\mathbf{A}}|&= \frac{d}{dx} \log \left( |{\mathbf{Q}}| \cdot |\varvec{{\Lambda }}| \cdot |{\mathbf{Q}}^{-1}| \right) = \frac{d}{dx} \log |\varvec{{\Lambda }}| = \frac{d}{dx} \sum _i \log \varvec{{\Lambda }}_{ii} \\&= \sum _i \frac{1}{\varvec{{\Lambda }}_{ii}} \frac{d {\varvec{\Lambda }}_{ii}}{dx} = {{\mathrm{tr}}}\left[ {\varvec{\Lambda }}^{-1} \frac{d {\varvec{\Lambda }}}{dx} \right] , \end{aligned} \end{aligned}$$

which is equal to the right-hand side of Eq. 60 since

62 $$\begin{aligned} \begin{aligned} {{\mathrm{tr}}}\left[ {\mathbf{A}}^{-1} \frac{d{\mathbf{A}}}{dx} \right]&= {{\mathrm{tr}}}\left[ {\mathbf{Q}} {\varvec{\Lambda }}^{-1} {\mathbf{Q}}^{-1} \frac{d {\mathbf{Q}}}{dx} {\varvec{\Lambda }} {\mathbf{Q}}^{-1} \right] + {{\mathrm{tr}}}\left[ {\mathbf{Q}} {\varvec{\Lambda }}^{-1} \frac{d {\varvec{\Lambda }}}{dx} {\mathbf{Q}}^{-1} \right] - {{\mathrm{tr}}}\left[ \frac{d {\mathbf{Q}}}{dx} {\mathbf{Q}}^{-1} \right] \\&= {{\mathrm{tr}}}\left[ \frac{d {\mathbf{Q}}}{dx} {\mathbf{Q}}^{-1} \right] + {{\mathrm{tr}}}\left[ {\varvec{\Lambda }}^{-1} \frac{d {\varvec{\Lambda }}}{dx} \right] - {{\mathrm{tr}}}\left[ \frac{d {\mathbf{Q}}}{dx} {\mathbf{Q}}^{-1} \right] = {{\mathrm{tr}}}\left[ {\varvec{\Lambda }}^{-1} \frac{d {\varvec{\Lambda }}}{dx} \right] . \end{aligned} \end{aligned}$$

## Appendix 2: Hessian approximation

For an appropriate model, it holds that

63 $$\begin{aligned} {\mathbf{E}}[\varvec{\epsilon }_{ij}] = \varvec{0}, \end{aligned}$$

and

64 $$\begin{aligned} {\mathbf{E}} \left[ \varvec{\epsilon }_{ij} \varvec{\epsilon }_{ij}^{T} \right] = {\mathbf{R}}_{ij}, \end{aligned}$$

where the expected values are taken with respect to data, which here are considered to be random variables whose values have not yet been realized. Based on these equations, the Hessian in Eq. 13 can be simplified to various degrees by approximating its different terms with their expected values. A minimal simplification for eliminating the second order derivative terms is achieved by noting that

65 $$\begin{aligned} {\mathbf{E}} \left[ 2 \varvec{\epsilon }_{ij}^T {\mathbf{R}}_{ij}^{-1} \frac{d^2 \varvec{\epsilon }_{ij}}{d \eta _{ik} d \eta _{il}} \right] = {\mathbf{E}} \left[ 2 \varvec{\epsilon }_{ij}^T \right] {\mathbf{R}}_{ij}^{-1} \frac{d^2 \varvec{\epsilon }_{ij}}{d \eta _{ik} d \eta _{il}} = 0, \end{aligned}$$

and

66 $$\begin{aligned} \begin{aligned}&{\mathbf{E}} \left[ - \varvec{\epsilon }_{ij}^T {\mathbf{R}}_{ij}^{-1} \frac{d^2 {\mathbf{R}}_{ij}}{d \eta _{ik} d \eta _{il}} {\mathbf{R}}_{ij}^{-1} \varvec{\epsilon }_{ij} + {{\mathrm{tr}}}\left[ {\mathbf{R}}_{ij}^{-1} \frac{d^2 {\mathbf{R}}_{ij}}{d \eta _{ik} d \eta _{il}} \right] \right] \\&\quad = {\mathbf{E}} \left[ - {{\mathrm{tr}}}\left[ \varvec{\epsilon }_{ij}^T {\mathbf{R}}_{ij}^{-1} \frac{d^2 {\mathbf{R}}_{ij}}{d \eta _{ik} d \eta _{il}} {\mathbf{R}}_{ij}^{-1} \varvec{\epsilon }_{ij} \right] \right] + {{\mathrm{tr}}}\left[ {\mathbf{R}}_{ij}^{-1} \frac{d^2 {\mathbf{R}}_{ij}}{d \eta _{ik} d \eta _{il}} \right] \\&\quad ={\mathbf{E}} \left[ - {{\mathrm{tr}}}\left[ {\mathbf{R}}_{ij}^{-1} \frac{d^2 {\mathbf{R}}_{ij}}{d \eta _{ik} d \eta _{il}} {\mathbf{R}}_{ij}^{-1} \varvec{\epsilon }_{ij} \varvec{\epsilon }_{ij}^T \right] \right] + {{\mathrm{tr}}}\left[ {\mathbf{R}}_{ij}^{-1} \frac{d^2 {\mathbf{R}}_{ij}}{d \eta _{ik} d \eta _{il}} \right] \\&\quad = - {{\mathrm{tr}}}\left[ {\mathbf{R}}_{ij}^{-1} \frac{d^2 {\mathbf{R}}_{ij}}{d \eta _{ik} d \eta _{il}} \right] + {{\mathrm{tr}}}\left[ {\mathbf{R}}_{ij}^{-1} \frac{d^2 {\mathbf{R}}_{ij}}{d \eta _{ik} d \eta _{il}} \right] = 0 ,\end{aligned} \end{aligned}$$

where we are making use of the fact that the trace of a scalar is just the scalar, the order of the expectation and trace operators can be shifted, and the cyclic property of the trace operator. This simplification is used in the present study.

Further simplifications of Eq. 13 may be performed by noting that the expectation of additional terms vanishes,

67 $$\begin{aligned}&{\mathbf{E}} \left[ - 2 \varvec{\epsilon }_{ij}^T {\mathbf{R}}_{ij}^{-1} \frac{d {\mathbf{R}}_{ij}}{d \eta _{il}} {\mathbf{R}}_{ij}^{-1} \frac{d \varvec{\epsilon }_{ij}}{d \eta _{ik}} \right] = 0,\end{aligned}$$

68 $$\begin{aligned}&{\mathbf{E}} \left[ - 2 \varvec{\epsilon }_{ij}^T {\mathbf{R}}_{ij}^{-1} \frac{d {\mathbf{R}}_{ij}}{d \eta _{ik}} {\mathbf{R}}_{ij}^{-1} \frac{d \varvec{\epsilon }_{ij}}{d \eta _{il}} \right] = 0, \end{aligned}$$

and by taking the expected value and collecting terms,

69 $$\begin{aligned} \begin{aligned}&{\mathbf{E}} \left[ 2 \varvec{\epsilon }_{ij}^T {\mathbf{R}}_{ij}^{-1} \frac{d {\mathbf{R}}_{ij}}{d \eta _{ik}} {\mathbf{R}}_{ij}^{-1} \frac{d {\mathbf{R}}_{ij}}{d \eta _{il}} {\mathbf{R}}_{ij}^{-1} \varvec{\epsilon }_{ij} - {{\mathrm{tr}}}\left[ {\mathbf{R}}_{ij}^{-1} \frac{d {\mathbf{R}}_{ij}}{d \eta _{il}} {\mathbf{R}}_{ij}^{-1} \frac{d {\mathbf{R}}_{ij}}{d \eta _{ik}}\right] \right] \\&\quad = {{\mathrm{tr}}}\left[ {\mathbf{R}}_{ij}^{-1} \frac{d {\mathbf{R}}_{ij}}{d \eta _{il}} {\mathbf{R}}_{ij}^{-1} \frac{d {\mathbf{R}}_{ij}}{d \eta _{ik}}\right] . \end{aligned} \end{aligned}$$

Taken together, all simplifications yield the following Hessian

70 $$\begin{aligned} \mathrm {\tilde {H}}_{ikl} = - \sum _{j=1}^{n_{i}} \Bigg ( \frac{d \varvec{\epsilon }_{ij}^T}{d \eta _{il}} {\mathbf{R}}_{ij}^{-1} \frac{d \varvec{\epsilon }_{ij}}{d \eta _{ik}} + \frac{1}{2} {{\mathrm{tr}}}\left[ {\mathbf{R}}_{ij}^{-1} \frac{d {\mathbf{R}}_{ij}}{d \eta _{il}} {\mathbf{R}}_{ij}^{-1} \frac{d {\mathbf{R}}_{ij}}{d \eta _{ik}}\right] \Bigg ) - \mathrm {\Omega }_{kl}^{-1}, \end{aligned}$$

which is the variant used in NONMEM [2].

## Appendix 3: Benchmark models and data

The equations for the two-compartment pharmacokinetic model are

71 $$\begin{aligned} \begin{aligned} V_1 \frac{d \, c_1(t)}{dt}&= u(t) + Cl_d \, (c_2(t) - c_1(t)) - \frac{V_{max} \, c_1(t)}{K_m \, c_1(t)} \\ V_2 \, \frac{d \, c_2(t)}{dt}&= Cl_d \, (c_1(t) - c_2(t)) \\ c_1(0)&= c_2(0) = 0, \end{aligned} \end{aligned}$$

where $u(t)$ is an input function, which was used to model a constant infusion with the rate 0.67 per minute during the first 30 minutes followed by another 30 minutes of washout. For models M1 and M2, the scalar-valued observation model was defined by ${\mathbf{y}}_t = c_1(t) + {\mathbf{e}}_t$, where ${\mathbf{e}}_t \in N(0,{\mathbf{R}}_t)$ and

72 $$ {\mathbf{R}}_{t}  = \left( {\begin{array}{*{20}c}    {r_{{a1}}^{2} }  \\   \end{array} } \right). $$

For models M3 and M4, the vector-valued observation model was defined by ${\mathbf{y}}_t = (c_1(t), c_2(t)) + {\mathbf{e}}_t$, where

73 $$  {\mathbf{R}}_{t}  = \left( {\begin{array}{*{20}c}    {(r_{{a1}}  + r_{{p1}} {\mkern 1mu} c_{1} (t))^{2} } & {}  \\    {} & {r_{{a2}}^{2} }  \\   \end{array} } \right). $$

In models M1 and M2, the three parameters $V_{max}$, $K_m$, and $V_1$, were defined to be log-normally distributed on the population level. This was accomplished by multiplying them with $\exp (\eta _1)$, $\exp (\eta _2)$, and $\exp (\eta _3)$, respectively, where $\varvec{\eta } = (\eta _1, \eta _2, \eta _3)$ is normally distributed with zero mean. In the first variant of this model, M1, the covariance matrix for the random effect parameters is defined by the diagonal matrix

74 $$   {\mathbf{\Omega }} = \left( {\begin{array}{*{20}c}    {\omega _{{11}}^{2} } & {} & {}  \\    {} & {\omega _{{22}}^{2} } & {}  \\    {} & {} & {\omega _{{33}}^{2} }  \\   \end{array} } \right), $$

and in the second variant, M2, the full matrix is estimated using the parameterization

75 $$ {\mathbf{\Omega }} = \left( {\begin{array}{*{20}c}    {\omega _{{11}}^{2}  + \omega _{{12}}^{2}  + \omega _{{13}}^{2} } & {\omega _{{12}} \omega _{{22}}  + \omega _{{13}} \omega _{{23}} } & {\omega _{{13}} \omega _{{33}} }  \\    {\omega _{{12}} \omega _{{22}}  + \omega _{{13}} \omega _{{23}} } & {\omega _{{22}}^{2}  + \omega _{{23}}^{2} } & {\omega _{{23}} \omega _{{33}} }  \\    {\omega _{{13}} \omega _{{33}} } & {\omega _{{23}} \omega _{{33}} } &{\omega _{{33}}^{2} }  \\   \end{array} } \right)$$

to ensure positive definiteness. In models M3 and M4, an additional random effect parameter was in the same way introduced for the parameter $Cl_d$. A similarly defined full matrix for 4 random effect parameters was used for models M3 and M4.

The parameter values used for simulating data are shown in Table 2, together with information of which parameters are being estimated in the four model variants, and what the starting values of the estimation were. One data set consisting of 10 simulated individuals was used for models M1 and M2. Here, the values of $c_1$ were collected at the time points $t=10,15,20,\ldots ,60$. For models M3 and M4, another data set consisting of 20 simulated individuals was used, where the values of $c_1$ and $c_2$ were collected at the time points $t=10,15,20,\ldots ,60$.

**Table 2:** Parameter values used for simulating data (D), starting values for estimation (S), and parameter estimates (E) for the different models

Parameter	D	S, M1	S, M2	S, M3/M4	E, M1	E, M2	E, M3	E, M4
$V_{max}$	0.5	0.2	0.2	0.2	0.424	0.419	0.473	0.473
$K_m$	4	3	3	3	3.91	2.53	4.37	4.37
$Cl_d$	0.01	–	0.01	0.01	–	0.00976	0.00813	0.00813
$V_1$	0.3	0.1	0.1	0.1	0.288	0.285	0.321	0.321
$V_2$	0.1	–	0.1	0.1	–	0.0956	0.0959	0.0959
$r_{a1}$	$\sqrt{0.5} \approx 0.707$	–	$\sqrt{0.1} \approx 0.316$	$\sqrt{0.1} \approx 0.316$	–	0.414	0.644	0.644
$r_{p1}$	0$^*$	−$^*$	−$^*$	$\sqrt{0.1} \approx 0.316$	−$^*$	−$^*$	0.00165	0.00163
$r_{a2}$	$\sqrt{0.5}^* \approx 0.707$	−$^*$	−$^*$	$\sqrt{0.1} \approx 0.316$	−$^*$	−$^*$	0.730	0.730
$\omega _{11}$	$\sqrt{0.5} \approx 0.707$	1	1	1	0.616	0.553	0.559	0.560
$\omega _{12}$	0	–	0	0	–	−0.0518	−0.123	−0.123
$\omega _{13}$	0	–	0	0	–	0.439	−0.138	−0.138
$\omega _{14}^*$	0$^*$	−$^*$	−$^*$	0	−$^*$	−$^*$	0.0273	0.0275
$\omega _{22}$	$\sqrt{0.5} \approx 0.707$	1	1	1	0.772	0.575	0.533	0.533
$\omega _{23}$	0	–	0	0	–	$-$0.485	0.0174	0.0174
$\omega _{24}^*$	0$^*$	−$^*$	−$^*$	0	−$^*$	−$^*$	−0.0230	−0.0230
$\omega _{33}$	$\sqrt{0.5} \approx 0.707$	1	1	1	0.994	1.39	0.776	0.776
$\omega _{34}^*$	0$^*$	−$^*$	−$^*$	0	−$^*$	−$^*$	−0.409	−0.409
$\omega _{44}^*$	$\sqrt{0.5}^* \approx 0.707$	−$^*$	−$^*$	1	−$^*$	−$^*$	0.870	0.870

Parameters which were not estimated are indicated with a dash. The * indicate that a parameter is only used in models M3 and M4
